# Rif1 promotes association of G-quadruplex (G4) by its specific G4 binding and oligomerization activities

**DOI:** 10.1038/s41598-019-44736-9

**Published:** 2019-06-13

**Authors:** Hisao Masai, Rino Fukatsu, Naoko Kakusho, Yutaka Kanoh, Kenji Moriyama, Yue Ma, Keisuke Iida, Kazuo Nagasawa

**Affiliations:** 1 Department of Genome Medicine, Tokyo Metropolitan Institute of Medical Science, Kamikitazawa, Setagaya-ku, Tokyo 156-8506 Japan; 2 Department of Biotechnology and Life Science, Faculty of Technology, Tokyo University of Agriculture and Technology, Tokyo, 184-8588 Japan; 3 Molecular Chirality Research Center, Synthetic Organic Chemistry, Department of Chemistry, Graduate School of Science, Chiba University, Chiba, 263-8522 Japan

**Keywords:** DNA, Chromosomes, Origin selection

## Abstract

Rif1 is a conserved protein regulating replication timing and binds preferentially to the vicinity of late-firing/dormant origins in fission yeast. The Rif1 binding sites on the fission yeast genome have an intrinsic potential to generate G-quadruplex (G4) structures to which purified Rif1 preferentially binds. We previously proposed that Rif1 generates chromatin architecture that may determine replication timing by facilitating the chromatin loop formation. Here, we conducted detailed biochemical analyses on Rif1 and its G4 binding. Rif1 prefers sequences containing long stretches of guanines and binds preferentially to the multimeric G4 of parallel or hybrid/mix topology. Rif1 forms oligomers and binds simultaneously to multiple G4. We present a model on how Rif1 may facilitate the formation of chromatin architecture through its G4 binding and oligomerization properties.

## Introduction

DNA replication proceeds in accordance with temporal and spatial program that is intrinsic to each cell type^1^. In higher eukaryotes, replication timing may be determined by megabase chromosome domains that dictate the temporal units for DNA replication^2,3^. Rif1, a conserved nuclear factor originally identified as a telomere binding protein in yeasts^4,5^, was recently shown to play a major role in defining the replication timing domains in both yeast^6,7^ and mammalian cells^8–10^.

In fission yeast *rif1*∆ cells, late/dormant origins are fired in the presence of HU (hydroxy urea) or fired early even during normal S phase and initiation at some early-firing origins was reduced^6^. In mammalian cells, replication timing domain structures were dramatically altered in Rif1-depleted or knockout cells^8–10^. It was shown that chromatin loop length became longer in Rif1-depleted cells, suggesting that Rif1 may be involved in generation of chromatin loop structures^8^. Immunostaining indicated that Rif1 is localized at nuclear periphery in Triton- and DNaseI-insoluble structures, showing that it associates with nuclear matrix structures, probably tethering chromatin fibers at nuclear periphery. It has been known that mid-to-late replication foci are localized at nuclear periphery^1,11^. Thus, we proposed that Rif1 may generate chromatin compartments that define mid-to-late replicating chromosome domains. Analyses of Rif1 binding sites and chromatin interactions by the 4C-seq assays in mouse ES cells also indicated that Rif1 confined the chromatin interactions within each replication timing domain^12^.

It was also reported that Rif1 carries binding sites for phosphatase (PP1; Protein Phosphatase 1)^13–18^ and that the recruited phosphatase inhibits initiation by antagonizing the phosphorylation events mediated by Cdc7 kinase. Therefore, Rif1’s abilities to organize functional chromatin domains and to recruit a phosphatase contribute to temporal and spatial regulation of DNA replication.

Mammalian Rif1 was not detected on normal telomeres^19^, but was implicated in cellular responses to replication stress^20–22^. It was also reported to suppresse homologous recombination-dependent repair by inhibiting an end-resection reaction and stimulate non-homologous end-joining repair^23–27^. Recent reports indicate additional roles of Rif1 at the replication forks and S phase regulation during early embryogenesis^28–30^. It would be an interesting possibility that Rif1’s ability to organize chromatin architecture at nuclear periphery may be involved also in regulation of these chromosome events.

Analyses of Rif1 binding sequences (Rif1BS) with ChIP-seq led to the identification of Rif1CS (Rif1 binding consensus sequence) containing 5–6 runs of guanine residues^31^. We then showed that Rif1BS can adopt G-quadruplex(G4)-like structures *in vitro* and the purified Rif1 protein binds to G4 structures. Strong correlation between the ability of Rif1BSs to form G4 *in vitro* and binding of Rif1 to these sequences *in vivo* indicates that Rif1 specifically recognizes G4 structures that are indeed formed in cells.

In order to clarify how Rif1 interacts with G4 DNA and contributes to the formation of specific nuclear architecture, we have conducted detailed analyses of target sequences of fission yeast Rif1 protein, and also biochemically characterized this protein. We found that Rif1 preferentially binds to multimeric G4 structures with parallel or hybrid/mix-type topology containing 5–6 runs of guanine and show that Rif1 protein forms oligomers and promotes association of multiple DNAs containing G4 structures. On the basis of theses data, we will present a model on how Rif1 may interact with G4 DNA and how it may contribute to the establishment of replication timing domains.

## Results

### Purification of the full-length fission yeast Rif1 protein

**Figure 1:**
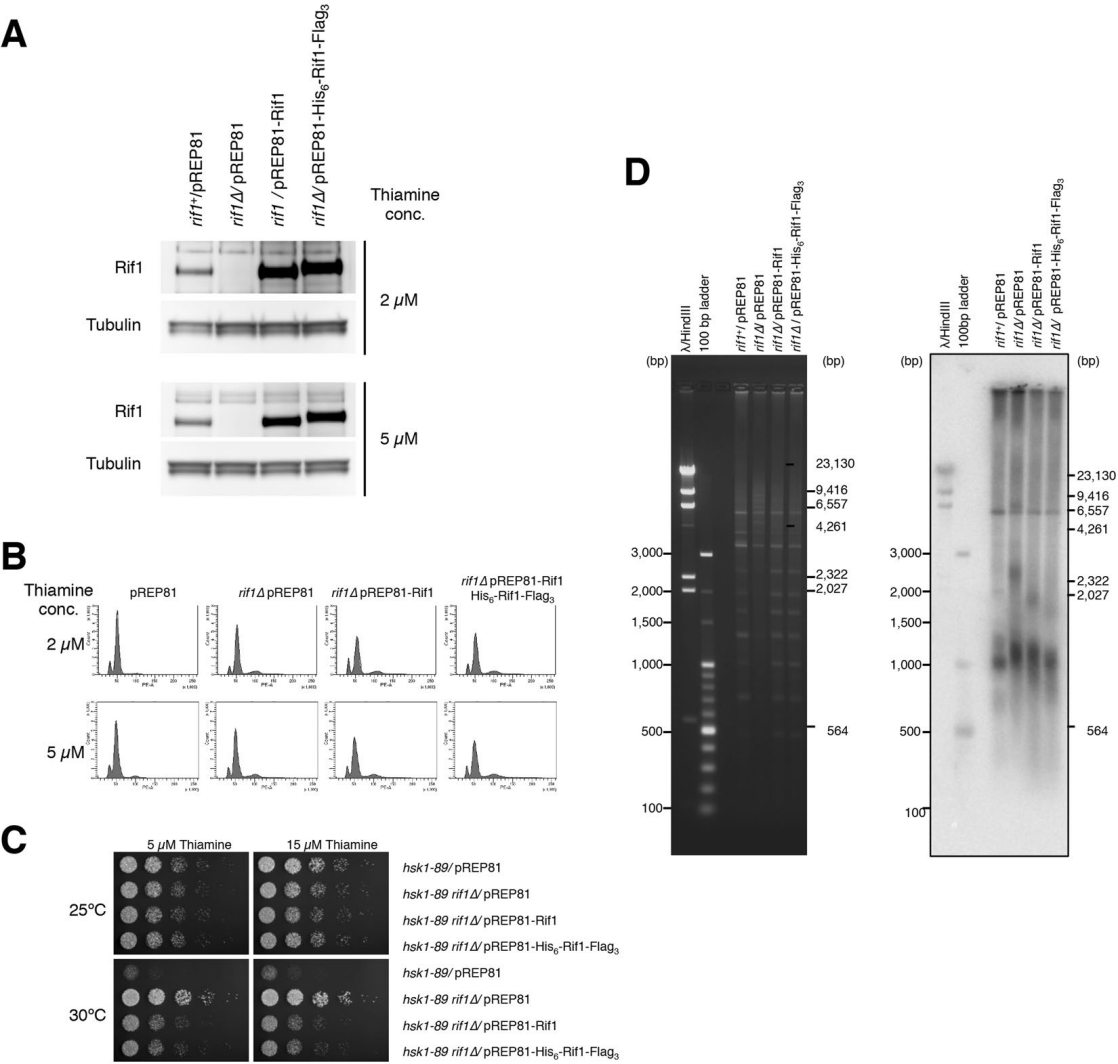
His_6_-Rif1-Flag_3_ protein is functional. (**A**,**B**) The wild-type or *rif1*∆ harboring pREP81 vector, or *rif1*∆ harboring pREP81-Rif1 or pREP81-His_6_-Rif1-Flag_3_ were grown in SD +2 µM thiamine, and cells were washed with EMM without thiamine and transferred to the EMM medium containing the indicated concentration (2 µM or 5 µM) of thiamine for 16 hrs. (**A**) The expression levels of Rif1 and tubulin were examined by western blotting. (**B**) Cell cycle was analyzed by FACS. (**C**) The 5-fold serial dilution of the cells, indicated in the figure, was spotted on EMM plate containing 5 or 15 μM thiamine. The plates were incubated at 25 °C or 30 °C. The photographs were taken at 4 days after plating. (**D**) Function in telomere length regulation. Genomic DNA was extracted from the cells indicated, digested with *Eco*RI and electrophoresed on 1.2% agarose in TAE buffer. The digested DNA was transferred to nitrocellulose membrane and fixed with UV irradiation, followed by hybridization with ^32^P-labeled telomere DNA. Left, EtBr-stained gel; right, Southern blotting of the same gel.

Fission yeast Rif1 (hereafter, referred to as “Rif1”; Rif1 from other species will be specified) protein is 1,400 amino acid long, composed of the N-terminal HEAT (Huntingtin, Elongation factor 3, A subunit of protein phosphatase 2A, and TOR) - and ARMADILLO-type repeats^13^ and a C-terminal unknown domain. We expressed the full-length Rif1 in human embryonic kidney cells 293T^32^ in an N-terminally His_6_ and C-terminally FLAG_3_-tagged form. We first showed that the presence of the tags at the N- and C-termini of the protein does not affect its function by showing i) expression of the tagged protein in ∆*rif1 hsk1–89*^ts^ abrogated the bypass of the Hsk1 function, and ii) the telomere length is not significantly affected by the tags. To assess the function of N-terminally His_6_- and C-terminally Flag_3_-tagged Rif1 protein, we expressed the His_6_-Rif1-Flag_3_ protein in *rif1*∆ cells. We took advantage of the fact that Hsk1 (the homologue of Cdc7 kinase) function is bypassed by *rif1* deletion. Expression of the functional Rif1 in *hsk1-89 rif1*∆ cells (viable at 30 °C) inhibits the growth of the strain at 30 °C (non-permissive for *hsk1-89*). This reflects the ability of Rif1 to inhibit the firing of late origins. We first examined the expression level of non-tagged Rif1 and His_6_-Rif1-Flag_3_ cloned on pREP81 in the presence of low levels of thiamine with western using the antibody that can detect the endogenous Rif1 protein. At 2 µM or 5 µM thiamine, both non-tagged and tagged proteins were expressed at, respectively, ~5 fold or ~3 fold more than the endogenous Rif1 protein (Fig. 1A). Cell cycle profiles of *rif1*∆ cells carrying pREP81 plasmid expressing non-tagged or tagged Rif1 exhibited the identical pattern (Fig. 1B). We then examined the effect of both proteins on the growth of *hsk1-89 rif1*∆ cells at 30 °C. *hsk1-89* harboring a vector can not grow at 30 °C, whereas *hsk1-89 rif1*∆ harboring the vector can grow at this temperature. *hsk1-89 rif1*∆ harboring pREP81-Rif1 grew poorly at 30 °C due to expression of the wild-type Rif1 protein. Similarly, *hsk1-89 rif1*∆ harboring pRPE81-His_6_-Rif1-Flag_3_ grew poorly (Fig. 1C). The extent of the growth was similar between non-tagged and tagged Rif1, indicating that His_6_-Rif1-Flag_3_ proteins retain the ability to inhibit the origin firing. The results were similar at both 5 µM and 15 µM thiamine.

We also examined the telomere functions of His_6_-Rif1-Flag_3_. The length of DNA fragments containing telomeres in the wild-type cells (YM71) was ~1,000 bp under our experimental condition, whereas that in *rif1*∆ cells was ~1,200 bp and an additional fragments were detected at around 2.5 kb. The telomere length returned to the wild-type level in *rif1*∆ cells harboring pREP81-His_6_-Rif1-Flag_3_, indicating that the double-tagged Rif1 retains the telomere regulation function as well (Fig. 1D).

**Figure 2:**
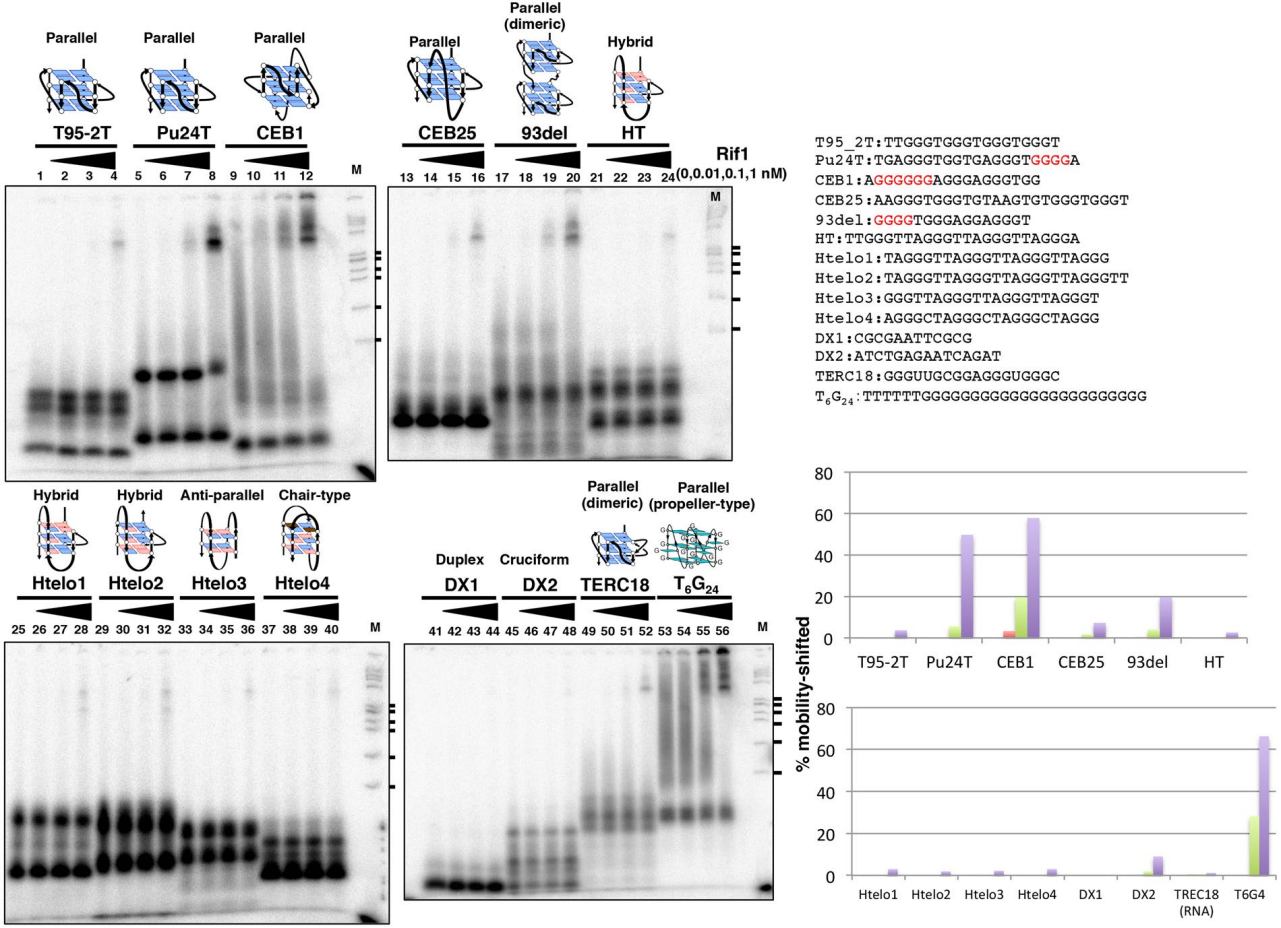
Binding of Rif1 to various single-stranded DNAs derived from sequences known to form specific G4 configuration. (**A**) Single-stranded oligonucleotide DNAs (0.2 pmole) as shown, which had been heat-denatured and renatured in 50 mM KCl and 40% PEG200, were incubated in the presence of increasing amounts of Rif1 protein (0, 0.1, 1 and 10 fmole [0, 0.01, 0.1 and 1 nM, respectively] of the Rif1 full-length polypeptide; the preparation used contains ~10-fold molar excess of degradation products [mainly a 70 kDa polypeptide derived from the C-terminus]) and were analyzed on 12% PAGE (0.5x TBE, 40% PEG200 and 50 mM KCl). List of oligonucleotides used in the assays and their sequences are shown. The schematic drawing of the structure of each G4 and its topology is shown, where known (see Supplementary Fig. S18 for the summary of the structures and references. The topologies of Htelo3 and Htelo4 were hybrid and anti-parallel, respctively, in our CD measurement [See Supplementary Figure S8].). The G-tracts longer than 3 in Pu24T, CEB1 and 93del are highlighted in red. The graph shows quantification of the Rif1 binding to each oligonucleotide. The values were calculated by dividing the radioactivity of the shifted bands (complex) by the sum of the free G4-structured DNA and the shifted bands (see also the Supplementary Fig. S1 and its legend). All the binding assays were conducted in separate experiments two times or more, with similar results, and only the representative data are presented. See Supplementary Fig. S2 for additional data related to this figure. M: molecular weight marker (ϕX174 DNA digested by *Hae*III). The ticks represent the sizes of 310, 271/281, 234, 194, 118 and 72 bp, from the top.

The protein was purified by consecutive anti-FLAG and nickel affinity columns. The protein is highly prone to degradation, giving rise to a 70 kDa truncated polypeptide, which is derived from the C-terminal segment (indicated by its reactivity to the C-terminal FLAG tag). In order to obtain a full-length form of Rif1 that is devoid of the 70 kDa degradation polypeptide, we conducted monoQ column fractionation or glycerol gradient fractionation, which permitted us to partially remove the degradation product (Supplementary Fig. S1A, lane 1 and data not shown). Using these preparations, we examined the affinity of the full-length Rif1 to a G4 substrate, T_6_G_24_ that forms a propeller-type parallel-stranded G4 containing three G-tetrad layers and three single-guanine propeller loops^33^ (Supplementary Fig. S1B; see a drawing in Fig. 2). The monoQ fraction contains ~1:2 ratio of the full-length and the 70 kDa polypeptide, and the apparent Kd of this preparation for this G4 DNA was ~0.3 nM (Supplementary Fig. S1C), when only the full-length polypeptide was considered. Even if the coexisting 70 kDa polypeptide (present at 4-fold molar excess than the full-length and on the assumption it binds to the substrate with affinity same as the full-length) were considered, the Kd would be less than 1.5 nM.

We examined the DNA binding property of the 70 kDa polypeptide. We were able to obtain the fractions containing only the 70 kDa polypeptide free from the full-length protein by glycerol gradient centrifugation or monoQ ion exchange chromatography (Supplementary Fig. S1A, lanes 2 and 3**)**. We conducted gel shift assays with these preparations along with the full-length fraction. The 70 kDa polypeptide exhibited much reduced affinity compared to the full-length; at 10 nM only a half of the substrate was bound (with estimated Kd > 10 nM; Supplementary Fig. S1B,C). Two different preparations of the 70 kDa polypeptide showed almost identical affinity to the G4 substrate. Thus, we can safely conclude that the full-length Rif1 protein binds to G4 DNA with extremely high affinity, while the 70 kDa polypeptide binds to the same DNA with at least one order of magnitude lower affinity. Furthermore, we can be reassured that our preparation of Rif1, although not completely devoid of the C-terminally derived polypeptide, represents mostly the binding of the full-length form at a lower concentration, and thus will be used in the following experiments.

### Interaction of Rif1 with various G4 DNAs

We examined the binding of Rif1 protein to single-stranded DNAs that are known to form G4 structures. Among those examined, Rif1 bound to Pu24T, CEB1 and 93del more efficiently than others (Fig. 2, lanes 6–8, 10–12 and 18–20). These single-stranded DNAs contain 4~6 runs of guanines, suggesting a possibility that consecutive guanines may facilitate the generation of the structures favored by Rif1. Indeed, the G_24_ oligonucleotide known to generate a parallel-type G4 structure^33^ is bound by Rif1 with high affinity (Fig. 2, lanes 53–56). Notably, Rif1 binds to telomere-derived sequence only very inefficiently (Fig. 2, lanes 21–40). The Kds for different G4 oligonucleotides were estimated to be 0.5~0.9 nM, values similar to that for T_6_G_24_ (Supplementary Fig. S2)

**Figure 3:**
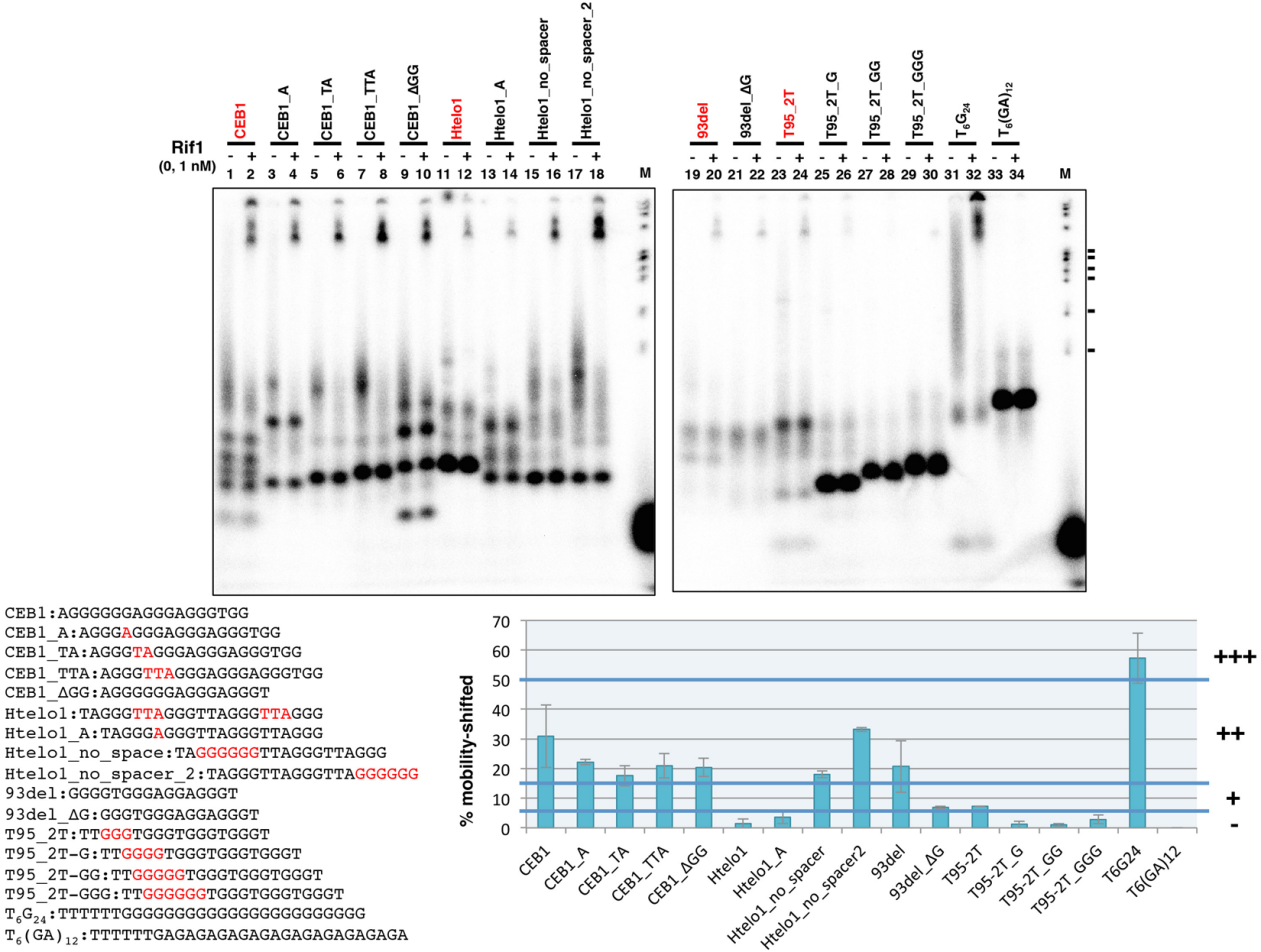
Binding of Rif1 to various single-stranded DNAs and their derivatives: effect of spacer sequence and runs of guanine on binding of Rif1. The single-stranded oligonucleotide DNAs (0.2 pmole) as shown, which had been heat-denatured and renatured in 50 mM KCl and 40% PEG200, were incubated in the absence (−) or presence (+) of Rif1 protein (10 fmole [1 nM] of the Rif1 full-length polypeptide; the preparation used contains ~10-fold molar excess of degradation products), and were analyzed on 12% PAGE (1x TBE, 50 mM KCl and 40% PEG200). List of oligonucleotides used in the assays and their sequences are shown below the panels. The relevant residues for modification are highlighted in red. The oligonucleotides from which derivatives were made are shown in red. The graph shows quantification of the Rif1 binding to each oligonucleotide with error bars, conducted as described in the legend to Fig. 2. All the binding assays were conducted in separate experiments two times or more with similar results, and one of the representative data are presented. The results of the same, but independent assays are shown in Supplementary Fig. S3. The oligonucleotides showing >50%, >16%, and >6% mobility-shift at 1 nM Rif1 were classified as +++, ++. + for Rif1 binding. M: molecular weight marker (ϕX174 DNA digested by *Hae*III). The ticks represent the sizes of 310, 271/281, 234, 194, 118 and 72 bp, from the top.

We then further examined the sequence preference of Rif1 by generating series of the derivatives of these good and poor binders. Htelo1 is not bound by Rif1 very efficiently (Fig. 2, lanes 25–28 and Fig. 3, lane 12). When the TTA spacer between the 5′-proximal two 3Gs was deleted (resulting in the generation of 6G near the 5′-end), binding efficiency was increased (Htelo1_no_spacer; Fig. 3, lane 16), while reducing the spacer to “A” did not affect the binding (Htelo1_A; Fig. 3, lane 14). Similarly, deletion of the TTA between the 3’-proximal two 3Gs (resulting in the generation of 6G at the 3′-end) led to even more increase of the Rif1 binding (Htelo1_no_spacer_2; Fig. 3, lane 18). On the other hand, insertions at the middle of 5′-proximal 6Gs did not significantly affect the Rif1 binding in CEB1 DNA, a relatively good binder (CEB1_A, CEB1_TA and CEB1_TTA; Fig. 3, lanes 1–8). Elongation of the 5′-proximal G runs in T95–2T did not increase, or even reduced the Rif1 binding (T95-2T-G, T95_2T_GG and T95_2T_GGG; Fig. 3, lanes 23–30), showing that simply the presence of a long G-tract is not sufficient for efficient Rif1 binding (see also Supplementary Fig. S3).

**Figure 4:**
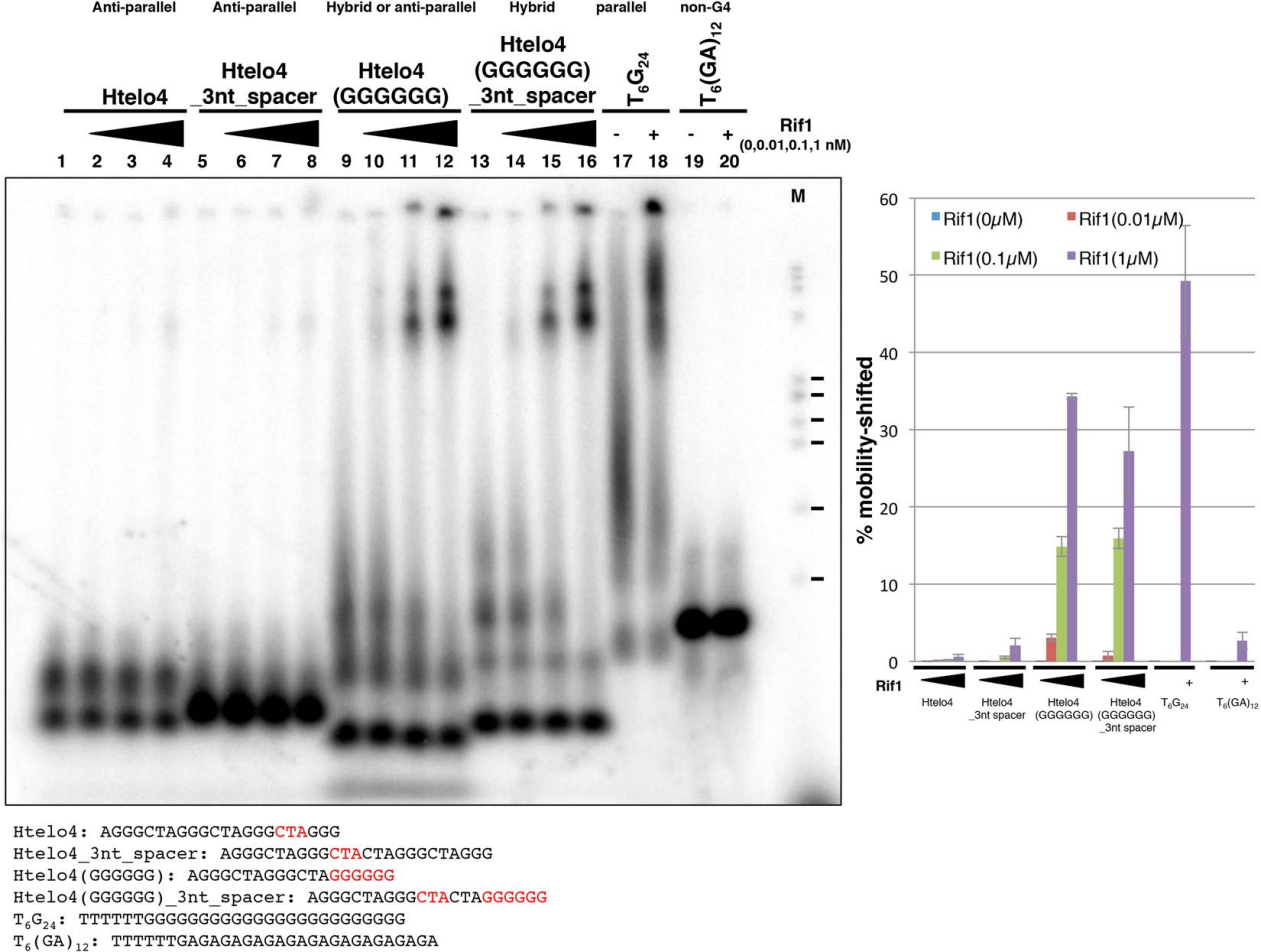
Binding of Rif1 to various single-stranded DNAs and their derivatives: stimulation of Rif1 binding by runs of guanine at the 3′-end of the DNA. The single-stranded oligonucleotide DNAs (0.5 pmole) as shown, which had been heat-denatured and renatured in 50 mM KCl and 40% PEG200, were incubated in the presence of increasing amounts of Rif1 protein. With T_6_G_24_ and T_6_(GA)_12_, 10 fmole (1 nM) of Rif1 was added (+). Samples were analyzed on 12% PAGE (1× TBE, 50 mM KCl and 40% PEG200). List of oligonucleotides used in the assays and their sequences are shown below the panels. The relevant residues for modification are highlighted in red. The topology, as determined by CD analyses (Supplementary Fig. S8), is indicated on top the lanes for each oligonucleotide. The graph shows quantification of the Rif1 binding to each oligonucleotide, conducted as described in the legends to Fig. 2. M: molecular weight marker (ϕX174 DNA digested by *Hae*III). The ticks represent the sizes of 310, 271/281, 234, 194, 118 and 72 bp, from the top. The quantification of the binding represents the average of three independent experiments with error bars. The background in the absence of Rif1 protein is subtracted. The results of the same, but independent assays are shown in Supplementary Fig. S4.

Htelo4 is also a poor binder of Rif1 (Fig. 2, lanes 37–40; Fig. 4, lanes 1–4), but deletion of CTA between the 3′-proximal two 3Gs significantly increased the binding (Htelo4(GGGGGG); Fig. 4, lanes 9–12). On the other hand, insertion of 3 nucleotides into the central loop did not affect the Rif1 binding both in the Htelo4 and Htelo4(GGGGGG) (Htelo4_3nt_spacer and Htelo4(GGGGGG)_3nt_spacer; Fig. 4, lanes 5–8 and 13–16; see also Supplementary Fig. S4). Both Htelo4_3nt_spacer and Htelo4(GGGGGG)_3nt_spacer show the presence of slow-migrating forms of DNA on PAGE which may represent oligomeric assembly of monomeric G4 or intermolecular G4 assembly. Rif1 preferentially binds to these slow-migrating forms. Indeed, those G4 oligonucleotides that are bound with Rif1 exhibit the slow migrating forms on polyacrylamide gel and those forms are preferentially bound by Rif1. In contrast, those that are poor binders generate very little slow migrating forms and predominantly generate a single band. Comprehensive analyses on polyacrylamide gel (12% PAGE with 10% PEG200) revealed that all the oligonucleotides bound by Rif1 showed slow-migrating forms (Supplementary Fig. S5). Thus, it appears that the ability to generate slow-migrating forms (representing oligomers of G4) may be a major determining factor for affinity of Rif to G4. The results also suggest that the presence of a long G-tract at the 3′-terminus may facilitate the formation of “multimeric” forms of G4 that are bound by Rif1 with higher affinity. The results also indicate that the loop length between the long 3′ G-tracts and the adjacent G-tracts does not significantly affect Rif1 binding.

### Conserved (A)GTGGG sequence in Rif1BS can mediate Rif1 binding

We previously identified Rif1CS (Rif1 consensus sequence; **CNWWGTGGGGG** [W = A or T]) through analyses of 35 Rif1BS sequences^31^. We also noted the presence of additional G-tracts in the vicinity of Rif1CS. Visual inspection of these sequences led us to realize that these additional G-tracts are frequently preceded by (A)GT, making (A)GTG_n_ (n = 3~6) a more plausible conserved sequence motif. We then synthesized (AGTGGG)_n_ (n = 1~4), (AGTGGGG)_n_ (n = 1~4), and (AGTGGGGG)_n_ (n = 1~4), and used them in gel shift assays to examine whether these conserved motifs are specifically recognized by Rif1. The oligonucleotides were heat-treated/renatured in 50 mM KCl and 40% PEG200 (a reagent that facilitates formation of G4 with molecular crowding effect^34^) before used in the assays. Among the oligonucleotides synthesized, the single “AGTGGG” was not bound with Rif1, but the single “AGTGGGG” or “AGTGGGGG” was weakly bound (Supplementary Fig. 6A, lanes 2, 8 and 14). The oligonucleotides carrying two or more repeats of AGTG_n_ were bound with Rif1. The most efficient binding was observed with three repeats (Supplementary Fig. S6A, lanes 6, 12 and 22 and Fig. S5b, lane 6).

We then synthesized (ACAGGGG)_n_ or (ACAGGGGG)_n_ (n = 1 or 3) to evaluate the significance of the conserved GT present before the G-tracts. Unexpectedly, Rif1 bound to ACAG_n_ oligonucleotides as efficiently as or slightly more efficiently than to the AGTG_n_ oligonucleotides (Supplementary Fig. S6B, compare lanes 2&4, 6&8, 10&12, and 14&16). The 8mer ACAGGGGG generated significant amounts of smeared slow-migrating forms after heat treatment, to which Rif1 bound (Supplementary Fig. S6B, lanes 11 and 12). The results indicate that these small G-rich oligonucleotides can form G4 structures which are preferred targets of Rif1 binding. We also examined whether G_6_, G_8_ and G_10_ tract oligonucleotide can be bound with Rif1, and showed that G_10_ can be bound with Rif1, and G_8_ to a small extent (Supplementary Fig. S7). Thus, the conserved AGT sequence before G-tracts does not appear to be crucial for Rif1 recognition, and it is less likely that the GTG_n_ sequence motif is specifically recognized by Rif1. However, we cannot rule out the possibility that the conserved GT sequence somehow facilitates the formation of a specific DNA structure on a duplex DNA that is preferentially bound by Rif1 in the cells.

### CD analyses of various G4-forming oligonucleotides suggest the preference of Rif1 for G4 with specific topology and increased stability

Various topologies are known to exist for G4 DNA. Those include parallel, anti-parallel, hybrid and mix^35^. These different topologies can be identified by measuring CD (Circular Dichroism) spectra^36–39^. In case of a typical anti-parallel structure, positive cotton effects are observed at 290 and 240 nm, and negative one at 265 nm. CD spectra of a typical parallel structure have positive and negative effects at 265 and 240 nm, respectively. In a typical hybrid structure, characteristic CD spectra are observed at 290 and 240 nm as positive and negative effects. We therefore measured the CD spectra of all the oligonucleotides used for Rif1 binding analyses (Supplementary Fig. S8 and Supplementary Table. S1). Htelo4, derived from telomeres, adopts anti-parallel forms and are not bound by Rif1 (Fig. 4, lanes 1–4). Generation of 6G stretch converted Htelo4 and its non-binding derivative (Htelo4_3nt_spacer; anti-parallel form) into good binders (Htelo4(GGGGGG) and Htelo4(GGGGGG)_3nt_spacer), which adopt hybrid forms (Fig. 4, lanes 9–16). However, Htelo1~2, which are judged to be hybrid forms, are poor binders (Fig. 2, lanes 25–32). Therefore, there may be other factors that converted the non-binders to the good binders. It could be the multimer formation by the latter DNAs (Supplementary Fig. S5). Other sequences that are bound by Rif1 are mostly parallel-type, but the derivatives of T95_2T (T95_2T_G, T95_2T_GG, and T95_2T_GGG) are parallel-type and are not efficiently bound by Rif1 (Fig. 3, lanes 25–30; T95_2T_GGG was weakly bound by Rif1, Supplementary Fig. S3, lane 30). Single-stranded DNA with sequences derived from Rif1BS are generally efficiently bound by Rif1^40^. Rif1–8, one of them, also adopts a parallel-type structure (Supplementary Fig. S8).

We next conducted melting assay to evaluate the stability of G4 by CD (Supplementary Fig. S9). In this assay, CD of T_6_G_24_, CEB1, CEB1_TA, CEB25, T95_2T and Htelo4 were measured at different temperatures (with 50 mM KCl). Structures of T_6_G_24_, CEB1, and CEB1_TA (good binders) were very stable, maintaining the parallel-type structure even at 95 °C. On the other hand, structures of CEB25, T95_2T (low efficiency binders) and Htelo4 (non-binder) are lost at this temperature. The structure of Htelo4 was most unstable, being disrupted at temperatures above 55 °C. In order to more precisely determine the melting temperatures of the stable G4, we conducted the melting assays at 10 mM KCl. Parallel-type structures were maintained for T_6_G_24_, CEB1, and CEB1_TA even under this condition. Structures of CEB25 and T95_2T were disrupted at higher temperatures (>75 °C for CEB25 and >85 °C for T95_2T). Our results suggest that there is general correlation between the heat stability and affinity to Rif1.

**Figure 5:**
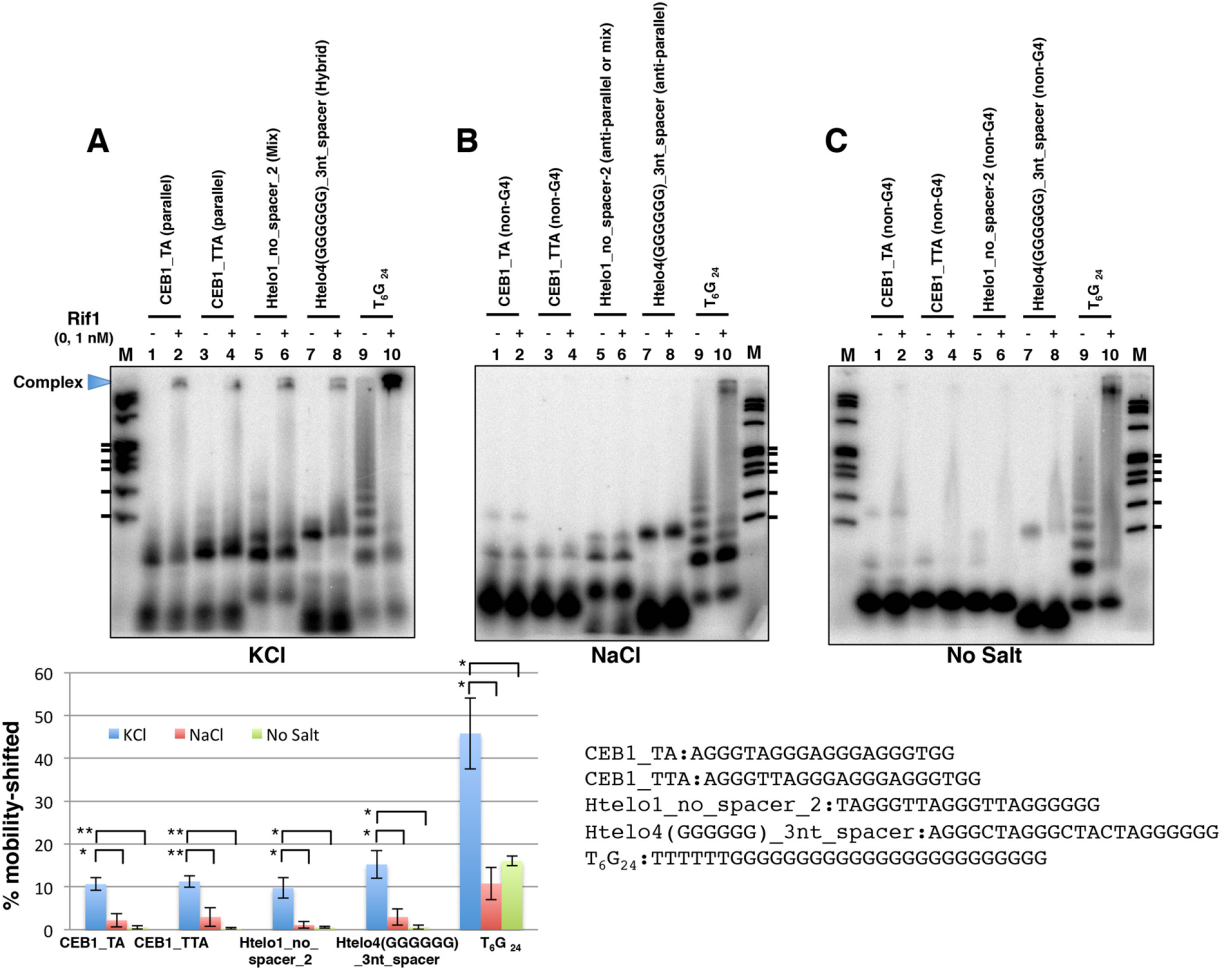
Effect of salt on Rif1 binding to selected G4 oligonucleotides whose topologies change in response to salt. The single-stranded oligonucleotide DNAs (0.25 pmole) as shown, which had been heat-denatured and renatured in 50 mM Tris-HCl (pH 7.5) with 50 mM KCl (**A**), 50 mM NaCl (**B**) or no salt (**C**), were incubated in the presence (+, 10 fmole [1 nM] of the Rif1 full-length polypeptide; the preparation used contains ~10-fold molar excess of degradation products as well) or absence (−) of Rif1 protein. Samples were analyzed on 8% PAGE (1x TBE, 10% glycerol) containing 50 mM KCl (**A**), 50 mM NaCl (**B**) or no salt (**C**). The Htelo1_no_spacer_2 and Htelo4(GGGGGG)_3nt_spacer adopt mix or hybrid-type topology, respectively, in KCl, and these forms are bound by Rif1 (**A**). However, they adopt anti-parallel type and are not efficiently bound by Rif1 in NaCl (**B**). The graph shows quantification of the Rif1 binding to each oligonucleotide, conducted as described in the legend to Fig. 2. M: molecular weight marker (ϕX174 DNA digested by *Hae*III). The ticks represent the sizes of 310, 271/281, 234, 194, 118 and 72 bp, from the top. The quantification of the binding represents the average of three independent experiments with error bars. The background in the absence of Rif1 protein is subtracted. *p < 0.1; **p < 0.05. The results of the same, but independent assays are shown in Supplementary Fig. S10.

Formation of G4 is affected by the presence of monovalent cations^41^, and it is known that the kind of salt affects the type of topology that a given sequence adopts. Indeed, the topology of some oligonucleotides changed from hybrid-type (in KCl) to anti-parallel (in NaCl). Therefore, we denatured/reannealed CEB1_TA, CEB1_TTA, Htelo1_no_spacer_2 and Htelo4(GGGGGG)_3nt_spacer in the presence of KCl or NaCl or in the absence of salt, and compared their affinity to Rif1. These DNAs were efficiently bound with Rif1 when they were heat denatured in the presence of KCl and PEG200 (Fig. 3, lanes 5–8, 17, 18; Fig. 4, lanes 13–16). In the presence of KCl, Rif1 bound efficiently to generate a high-molecular-weight complex on all the DNAs tested, in spite of the absence of PEG200 in the running gel (Fig. 5A). In contrast, in the absence of salt, all but T_6_G_24_ exhibited very little or much reduced mobility-shift upon heat denaturation and showed only inefficient binding with Rif1. T_6_G_24_, that can adopt a parallel form even in the absence of salt (Supplementary Fig. S8), was mobility-shifted upon heat denaturation and showed binding (Fig. 5B, lanes 9 and 10). In the presence of NaCl, CEB1_TA and CEB1_TTA showed very little mobility shift after heat treatment, consistent with non-G4 structures indicated by the CD profiles (Supplementary Fig. S8), and exhibited much reduced binding with Rif1 (Fig. 5B, lanes 1–4). On the other hand, Htelo1_no_spacer_2 and Htelo4(GGGGGG)_3nt_spacer was significantly mobility-shifted upon heat denaturation in NaCl, suggesting that some structures, most likely anti-parallel G4 structure as speculated from the CD results (Supplementary Fig. S8), are formed. However, there was only a low level of binding of Rif1 to these structures (Fig. 5C, lanes 5–8; see also Supplementary Fig. S10). These results supported our above speculation and indicated that the topology of G4 affects the efficiency of Rif1 binding probably through the formation of oligomeric G4s.

### Rif1 may promote association of G4-containing DNAs

**Figure 6:**
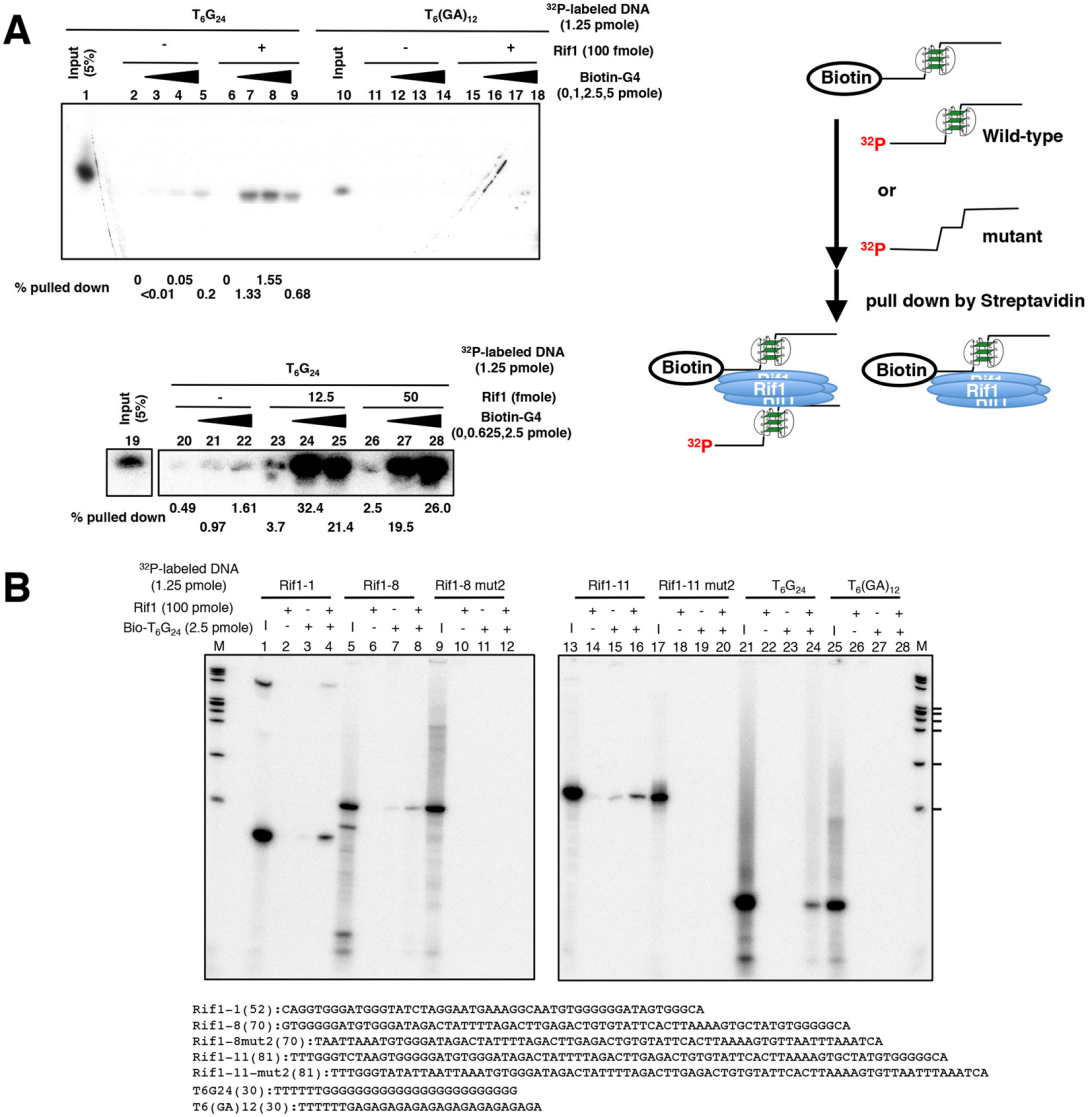
Rif1 can facilitate association of G4 DNA molecules. (**A**) Increasing amounts of biotin-labeled T_6_G_24_ DNA (denatured and renatured in 50 mM KCl and 40% PEG200; lanes 2, 6, 11 and 15, no biotin-labeled T_6_G_24_; lanes 3, 7, 12 and 16, 1 pmole; lanes 4, 8, 13 and 17, 2.5 pmole; lanes 5, 9, 14 and 18, 5 pmole) were mixed with ^32^P-labeled DNA templates (1.25 pmole [denatured and renatured in 50 mM KCl and 40% PEG200]; lanes 1–9, T_6_G_24_; lanes 10–18, T_6_[GA]_12_) in the presence (lanes 6–9 and 15–18) or absence (lanes 2–5 and 11–14) of Rif1 protein (100 fmole). Biotin-labeled DNA was pulled down by streptavidin beads, and washed with buffer containing 1 M NaCl before resuspended in formamide dye and boiling. Lanes 1 and 10, 5% of the input ^32^P DNA. Note that exactly same amount of ^32^P-labeled T_6_G_24_ and T_6_[GA]_12_ DNA was used. However, the extent of ^32^P end-labeling was four times less efficient with T_6_[GA]_12_ DNA than with T_6_G_24_ for some unknown reason. Lower panels show another set of experiments in which Biotin-labeled T_6_G_24_ (0, 0.625 and 2.5 pmole) was added in the presence or absence of Rif1 protein, as indicated in the figure. The pulled down materials were washed with binding buffer. Fractions of the pulled down materials relative to the input are indicated under each lane. The drawings (right) schematically represent the procedure of the experiments. (**B**) Pull-down of biotin-labeled T_6_G_24_ DNA (2.5 pmole) by streptavidin beads was conducted in the presence of various ^32^P-labeled DNA templates (1.25 pmole), as indicated. Rif1 protein (100 fmole) was also present, where indicated (+). The pulled down materials were washed with 1M NaCl before resuspended in formamide dye and boiling. I: 5% of the input ^32^P DNA. M: molecular weight marker (ϕX174 DNA digested by *Hae*III). The ticks represent the sizes of 310, 271/281, 234, 194, 118 and 72 nt, from the top. In both (**A**,**B**), the pulled down ^32^P-labelled DNAs were analyzed on 12% PAGE containing 8M urea (in 0.5x TBE).

We previously proposed that Rif1 may regulate chromatin architecture by facilitating chromatin loop formation^8,10^. Rif1 may hold together chromatin fibers by linking multiple G4 sequences. To explore this possibility, we examined if two G4 DNAs can be simultaneously bound by Rif1 protein. We have generated a biotin-labeled T_6_G_24_ oligonucleotide, which forms a parallel-type G4 structure^30^ (Supplementary Fig. S8) and is efficiently bound by Rif1 (Fig. 2, lanes 53–56; Fig. 3, lanes 31 and 32). We mixed this oligonucleotide with ^32^P-labeled T_6_G_24_ or T_6_[GA]_12_ (incapable of forming a G4 structure and not bound by Rif1, see lane 34 in Fig. 3) in the presence or absence of Rif1 protein, pulled down the biotin-T_6_G_24_ DNA with streptavidin-beads and examined whether ^32^P-labeled DNA is associated with the biotin-T_6_G_24_ DNA. We found that, in the presence of Rif1 protein, 1.55% of the input ^32^P-labeled T_6_G_24_ DNA was co-pulled down (Fig. 6A, lane 8), while ^32^P-labeled T_6_[GA]_12_ (not capable of forming G4) was not pulled down under the same condition (Fig. 6A, lanes 16–18). A small amount (0.2%) of ^32^P-labeled T_6_G_24_ DNA was pulled down even in the absence of Rif1 protein (Fig. 6A, lane 5), suggesting that the G4 DNA can self-associate with each other. This was conducted by washing the pulled down materials with 1 M NaCl. When the pulled down materials were washed by binding buffer (50 mM KCl) alone, close to 30% of the input ^32^P-labeled G4 DNA was pulled down by biotin-labeled G4 DNA. These results indicate that Rif1 can promote association of G4 molecules. It is not clear whether this is due to spontaneous disruption and regeneration of multimeric G4 between biotin-T_6_G_24_ and ^32^P-T_6_G_24_ or to association between pre-formed G4-structured T_6_G_24_ molecules.

The sequences derived from Rif1BS can form G4 structure^31^ and they can also be pulled down with biotin-T_6_G_24_ in the presence of Rif1 (Fig. 6B, lanes 4, 8, 16 and 24). Again, weak association of ^32^P-labeled DNA was observed even in the absence of Rif1 (Fig. 6B, lanes 3, 7 and 15). This association is dependent on the presence of G4 structure, since it is not observed with mutant forms of ^32^P-labeled DNA that do not form a G4 structure (Fig. 6B, lanes 12, 20 and 28). Thus, G4 molecules have potential of self-association.

### Rif1 forms oligomers

**Figure 7:**
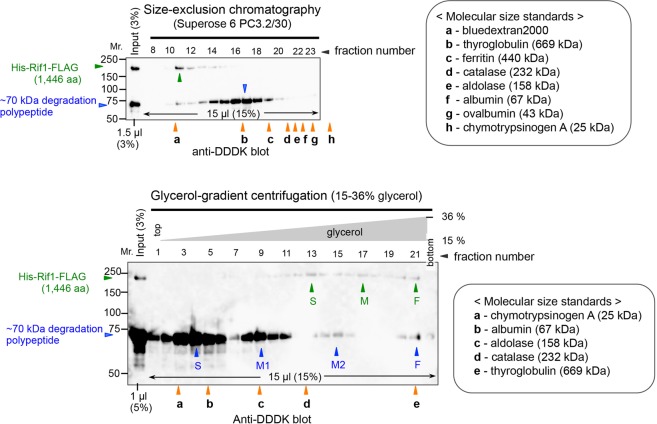
Physicochemical analyses of oligomeric states and molecular shapes of Rif1 polypeptides. Upper: Size-exclusion (gel-filtration) patterns of the partially purified fraction of His_6_-Rif1-FLAG_3_ (containing a ~70 kDa degradation product derived from the C-terminal segment). Retention volume of the molecular size standard was determined by monitoring A_260_. Lower: Sedimentation pattern of the same fraction through glycerol-gradient centrifugation. The three peaks of the full-length Rif1 are marked as F, M and S in order of sedimentation speed. The four peaks of the ~70 kDa polypeptide are marked similarly as F, M2, M1 and S. Sedimentation pattern of molecular size standards was analyzed by SDS-PAGE followed by silver-staining (data not shown). In both panels, the positions of size markers and those of the full-length and degradation polypeptide are shown by red, green and blue *filled arrowheads*, respectively.

The ability of Rif1 protein to facilitate the association of G4 structures could result from its potential to form oligomers. The potential oligomerization domains have been identified in the C-terminal segment of mammalian and yeast Rif1 proteins^22,42,43^. We have analyzed the fission yeast Rif1 protein in size-exclusion chromatography and glycerol gradient centrifugation (Fig. 7 and Supplementary Fig. S11). The full-length Rif1 migrated at three different positions (S, M and F) in glycerol gradient, with estimated subunit compositions of 4, 6, 8 deduced by one method (the Siegel and Monti method)^44^ or 8 and over 10 by another method (the Erickson method)^45^, although it was difficult to make precise estimation due to its extremely large size. The 70 kDa degradation polypeptide, derived from the C-terminal segment, migrates at four positions (S, M1, M2 and F) in glycerol gradient, and its subunit composition was estimated to be 2,4, 8 and 12. The results indicate that the Rif1 protein exists as various oligomeric forms with a highly elongated shape and suggest a possibility that the oligomeric Rif1 holds together chromatin fibers by binding to multiple G4 sequences through its subunits, promoting their association.

### Rif1 may be recruited to telomeres through different mechanisms

It has been known that Rif1 is recruited to telomere in a manner dependent on Taz1, another telomere binding protein^5^. We have examined whether Taz1 stimulates interaction of Rif1 with telomere sequence. For that, we first expressed the full-length Taz1 protein in mammalian cells and purified it (Supplementary Fig. S12A). We then used it to examine if it can facilitate the interaction of Rif1 with a typical telomere sequence, GGGTTA. Htelo3, containing repeats of GGGTTA and adopting an anti-parallel G4 (Supplementary Fig. S8**)**, was not bound by Rif1 very efficiently, as was shown above (Fig. 2, lanes 33–36). We added increasing amount of Taz1 in the absence or presence of Rif1. Taz1 also did not show significant binding to Htelo3 on its own. The presence of both proteins only showed additive complex formation (Supplementary Fig. S12B).

We then generated a 21 bp duplex DNA containing repeats of GGTTAC, the fission yeast telomere consensus sequence, and used this DNA as a substrate. Although Rif1 did not bind to this DNA on its own, Taz1 did with Kd of ~30 nM, consistent with the *in vivo* interaction of Taz1 with the telomere. Addition of increasing amount of Rif1 in the presence of Taz1 only slightly increased the amount of the shifted band (Supplementary Fig. S12C,D). These results indicate that Taz1 indeed directly binds to the double-stranded telomeric repeat sequences, consistent with the previous report with *in vitro* translated Taz1 protein^46^, but the efficient recruitment of Rif1 to telomere may require some additional factors or telomere chromatin structure.

## Discussion

Rif1 is a conserved nuclear protein that appears to play a major role in determining replication timing in both yeast and mammalian cells^6–10^. In the *rif1*∆ mutant of fission yeast, 29.6% of late/dormant origins (189 out of 638) are fired early in S phase^6^. In yeast, Rif1 does not affect pre-RC formation but inhibits the loading of Cdc45, the step regulated by Cdc7 kinase. Rif1 binding is enriched around the late/dormant origins on the fission yeast genome, suggesting that Rif1 binding somehow suppresses the pre-RC activation. In mammalian cells, depletion of Rif1 led to the increased level of Mcm phosphorylation mediated by Cdc7 kinase^8^. Rif1 was shown to recruit phosphatase both in yeast and human cells, thereby counteracting the phosphorylation of Mcms catalyzed by Cdc7 kinase^14–18^, providing explanation for these earlier observations. It was recently reported that Rif1 facilitates pre-RC formation by protecting ORC1 from degradation^17^. We proposed that Rif1 may regulate replication timing by generating the specific chromatin compartments through regulating the chromatin loops^8,10^. This prediction was supported by the fact that Rif1 binding to chromatin affects the replication initiation over close to 100 kb segment^31^. Analyses by 4C-seq indeed showed that mouse Rif1 regulates chromatin interactions within and between the replication domains^12^.

In yeasts, Rif1 not only binds to telomere but also to selected segments on the chromosome arms^6,47,48^. Genome-wide analyses of Rif1 binding sites and subsequent analyses of the binding sequences revealed the presence of a conserved G-rich sequence^31^. Further analyses showed the potential of these sequences to form G4-like structures and specific binding of Rif1 to these non-B DNA structures^31,40^. In mammalian cells, Rif1 binding is enriched in late-replicating domains, overlapping with LAD (Lamin-associated domains)^12^. Mammalian Rif1 was reported to bind to replication fork and Holliday junction DNA^22^ or to cruciform structures with high affinity^49^. Our analyses indicate that mouse Rif1 also preferentially binds to G4 structures *in vitro*^50^, and a subset of strong Rif1 binding sites on the chromatin are associated with G4-forming sequences (Moriyama, Yoshizawa and Masai, unpublished data). Diverse structures and biological functions of G4 DNA as well as their interacting proteins have been a focus of recent intense researches^51–54^. In this report, we have conducted detailed analyses on the specificity of G4 recognition by fission yeast Rif1 and biochemically characterized this protein. The results suggest a model on how Rif1 may regulate chromatin architecture through its ability to bind simultaneously to multiple G4 and to oligomerize.

### Rif1 prefers to bind to oligomeric assembly of G4, the formation of which is potentially facilitated by long runs of guanines

Various single-stranded DNAs known to form G4 structures were examined for their affinity to Rif1. Interestingly, Rif1 did not bind efficiently to telomere-derived sequences (repeats of GGGTTA; Figs 2, 3 and 4). Analyses of derivatives of the non-binders and good binders showed that the presence of long runs of guanine (6G) may be important but the length of the loop may not significantly affect the binding affinity (Figs 3 and 4). However, the presence of 6G is not sufficient for generation of the structure bound by Rif1, since insertion of 3G at one of the GGGT repeats (generating GGGGGGT near the 5′-terminus; T95-2T-GGG) did not improve the binding (Fig. 3). In contrast, generation of 6G at the 3’-terminus converted poor binders, Htelo1 and Htelo4, to good binders, Htelo1_no_spacer_2 and Htelo4(GGGGGG), respectively (Figs 3 and 4). Rif1-8, the 70 nt sequence derived from Rif1BS_I:4255_, formed ladders on PAGE, and these forms were preferentially bound by Rif1^40^ (Supplementary Fig. S13 and data not shown). This sequence contains a G5-tract near its 3′ end. Thus, the presence of long runs of guanines near the 3’-terminus could be important for generating structures preferred by Rif1 but its location and other sequence context also affects the affinity to Rif1. This conclusion is supported by the presence of 5 or 6 runs of guanine in all the Rif1CS, which appear at least twice within the high-affinity Rif1BS^31^.

We analyzed the numbers of Gs in the G-tracts (n = 3 or longer) on Rif1BS on both strands. We aligned the 35 Rif1BS in order of binding efficiency (determined by the intensities of the ChIP peaks) and counted the numbers of Gs in G_n(n=3 or longer)_ in the top 10 and bottom 10 binders. On the G-rich strand, 43.3% and 37.7% were 4G or longer, in the top10 and bottom10 Rif1BS, respectively. On the other hand, on the C-rich strand, 22% and 14.3% were 4G or longer in the top10 and bottom10, respectively (Supplementary Fig. S14). These data show that long G-tracts are enriched on the G-rich strand of Rif1BS, and corroborate the above *in vitro* results on Rif1-G4 interactions, showing that long G-tracts would constitute an element required for efficient Rif1 binding.

We also noted that Rif1 selectively binds to the slow-migrating forms generated by heat treatment, but not to the fast-migrating forms of the G4-forming single-stranded DNA, suggesting that Rif1 preferentially binds to the G4 assembly composed of multiple G4-forming sequences or multimerized G4 structures. It has been known that monomeric intramolecular quadruplexes, such as that formed by human telomeric DNA and RNAs, can dimerize by stacking end-to-end. More recently, sequences from the promoter regions of c-kit2 and B-raf or those from an intron of the N-myc gene have been shown by NMR analyses to generate G4 dimers^55,56^. In these cases, two strands are intertwined, each spanning the entire length of the structures, generating dimeric structures with six or seven consecutive G quartets. It is possible that similar dimeric or oligomeric structures are generated on the Rif1BS-derived sequences which carry multiple long G-tracts.

Those forms that are efficiently bound by Rif1 are generally slow migrating and often appear as smeared bands on PAGE, suggesting that the structures may be oligomers or intermolecular G4 structures. They may not be very stable (partially disrupted during the run on PAGE), or more dynamic than anticipated. Under selective gel electrophoresis conditions, both T_6_G_24_ and Rif1-8, very good binders of Rif1, generate clear ladders of molecules, each of which probably represents a distinct oligomeric form. The ladders are seen even on denaturing polyacrylamide gel after heat denaturation in formamide (Supplementary Fig. S13 and lane 25 of Supplementary Fig. S16), suggesting that DNA molecules may be composed of intertwined DNA strands indicative of interstrand G4 oligomers. Rif1 binding was observed also on simple G-tract sequences, such as 8G and 10G, albeit at a low level, suggesting the ability of Rif1 to interact with interstrand G4 (Supplementary Fig. S7). On the other hands, generation of oligomers through stacking of monomeric forms of intramolecular G4 is also possible, given the self-associating ability of G4 (Fig. 6). CD analyses of these single-stranded DNA suggested that topology of G4 may not be a sole determinant for Rif1 recognition (Supplementary Fig. S8), although the anti-parallel form may be least preferred by Rif1. This is most clearly shown by the fact the same oligonucleotide adopting different topology under different chemical conditions exhibits differential affinity to Rif1 (higher affinity in “hybrid” conformation than in “mix/anti-parallel” conformation; Fig. 5, Supplementary Fig. S10 and see also Supplementary Table S1).

### Rif1BS contains multiple copies of (A)GTG_n_ to which Rif1 can bind

Although the presence of long G-tracts characterizes Rif1BS, we noted the frequent occurrence of other G-tracts in the vicinity of Rif1CS. Furthermore, these G-tracts (3G or longer) are very frequently preceded by (A)GT. Analyses of the frequency of the dinucleotides preceding G_n(n=3 or longer)_ within the 1 kb segments surrounding the 35 Rif1BS showed that 42% was GT. This bias was observed only on the G-rich strand on which Rif1CS-G-tract is present, but not on the other C-rich strand (Supplementary Fig. S15).

Rif1 binds to oligonucleotides containing repeats of AGTG_n(n=3 or longer)_ and even to a single copy of AGTGGGG or AGTGGGGG that has been heat-treated. However, these bindings do not depend on the presence of GT, and ACA_n_ was also bound by Rif1 with similar or better efficiency, showing that sequence GT itself may not be an important determinant for Rif1 recognition *in vitro*. Indeed, Rif1 binds to G10 and to G8, to a small extent (Supplementary Fig. S7). The functional significance of this motif needs to be evaluated in the future by mutating the sequences on the genome and examining its effect on Rif1 binding and timing regulation in cells.

### Rif1 may promote association of multiple G4 DNAs

Biotinylated T_6_G_24_ can pull down ^32^P-labeled T_6_G_24_ DNA in the presence of Rif1 protein (Fig. 6). Thus, these experiments indicate the simultaneous binding of Rif1 to multiple DNA molecules. Analyses of Rif1 protein in size exclusion chromatography and glycerol gradient centrifugation indicate formation of oligomers composed of 4 to over 10 protomers (Fig. 7 and Supplementary Fig. S11). The G4 oligonucleotides used in this study, including T_6_G_24_, form G4 structures even without heat treatment (data not shown), suggesting that it is not likely that Rif1 facilitates the G4 formation, and our FRET experiments show no evidence for Rif1-mediated stabilization of G4 (Masai, Kanoh and Kakusho, unpublished data). These results support our conclusion that Rif1 promotes association of multiple G4 DNAs rather than facilitating the formation of G4 or stabilizing G4.

**Figure 8:**
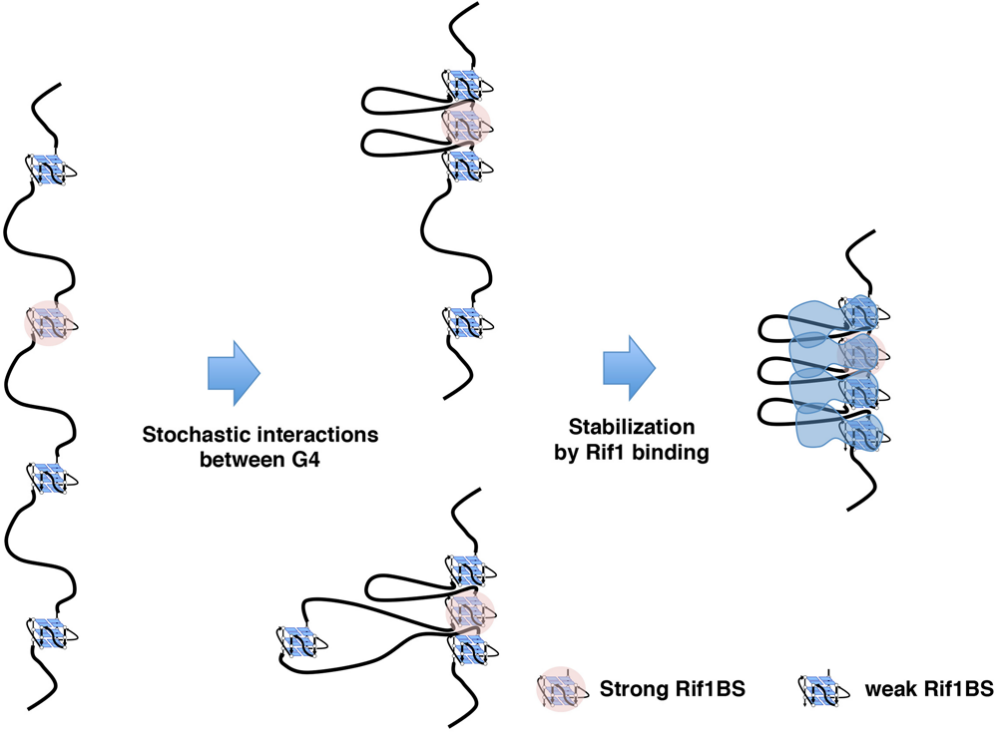
A model on actions of G4 and Rif1 in chromatin organization. G4 structures generated on the genomes may stochastically interact with each other through their self-association ability. A strong binder of Rif1 (indicated by pink circles), which probably forms more stable G4, would be recognized and bound by Rif1. This may stabilize the transient interactions between multiple G4s through its G4 binding and oligomerization abilities, leading to formation of chromatin compartment related to replication timing. The length of chromatin loop in the drawing is not to the actual size, which should be much larger.

We propose that the oligomeric Rif1 may tether multiple chromatin fibers through each subunit binding to different G4, potentially contributing to the formation of chromatin loops in the cells. This process would probably be facilitated by the ability of the N-terminal HEAT repeat segments to cooperatively spread on DNA and to encase DNA^57^. We speculate that the formation of chromatin loops may be dynamic, dictated by transient and even stochastic interaction of Rif1 and G4 (Fig. 8). Unexpectedly, we found that G4 self-associates *in vitro* even in the absence of Rif1. Indeed, biotinylated T_6_G_24_ can pull down not only heat-treated T_6_G_24_ but also Rif1BS-derived single-stranded DNA containing Rif1CS capable of forming G4 structures (Fig. 6B). The mutated forms of Rif1BS DNAs are not pulled down, showing that the association is specific to G4-like structures. This interaction is further enhanced by the presence of Rif1 (Fig. 6). Thus, an alternative, intriguing possibility is that chromatins make dynamic and stochastic interactions with each other through G4 structures present on the genome, and Rif1 may stabilize or facilitate these interactions (Fig. 8). Loss of a specific Rif1 binding site by mutation did not affect the binding of Rif1 to other binding sites including those close to the mutated binding site, as examined by ChIP-seq^31^, which could suggest that the inter-G4 interactions, if any, may be weak or transient.

In ChIP-chip analyses, we initially identified more than 160 Rif1 binding sites^6^. In ChIP-seq analyses, we first identified more than 90 Rif1BS, and reduced the numbers to 35 by selecting only the very reliable strong signals^31^. Since formation and stability of G4 structures are affected by the nucleotide sequence as well as by the microenvironment (salt concentration, transcriptional state *etc*.), there may be many unstable or transient G4 structures on the chromosomes.

We propose that Rif1 may generate a very dynamic and even stochastic chromatin domains. Rif1 bound to a strong site may serve as a hub with which surrounding chromatins interact in a transient and stochastic manner, and this may result in generation of a replication timing domain (Fig. 8). This model fits with recent reports on the effect of Rif1 on chromatin interactions in mammalian cells^12^. It was reported that *S. cerevisiae* Rif1 is tethered to nuclear membrane through its palmitoylation^58^. Since the late replicating domains are normally associated with the nuclear periphery, it is of interest whether similar membrane tethering contributes to the Rif1’s ability to generate specific chromatin architecture near the nuclear membrane in fission yeast and higher eukaryotes.

All the binding assays in this study have been conducted on G4 structures generated on single-stranded DNAs. However, *in vivo* situations are vastly different. G4 on Rif1BS are generated in a competition with the duplex structure which is more stable. It could be generated during the course of transcription or under the regional topological stress or epigenome modifications. Other proteins, such as Taz1, may affect chromatin interaction of Rif1. Indeed, Rif1 is recruited to telomere through Taz1^5^, probably not through direct DNA binding, and this is consistent with the low affinity of Rif1 to telomere-derived G4 (Figs 2, 3, 4 and Supplementary Fig. S12). Therefore, there may be multiple pathways for chromatin loading of Rif1 in cells. It would be necessary to analyze the G4 structures generated on a duplex Rif1BS and its interaction with Rif1. It would also be critically important to investigate the *in vivo* structures of Rif1BS DNAs, to demonstrate that G4 is generated at Rif1BS on the chromatin in cells, and to clarify molecular mechanisms of G4 formation in the cells as well as other unknown pathways for chromatin loading of Rif1.

## Materials and Methods

### Antibodies

The primary antibodies used were M2 (Sigma-Aldrich, F1804) and anti-DDDDK tag MAb (MBL, FLA-1).

### Expression and purification of fission yeast Rif1 protein and its derivatives

His_6_- and FLAG_3_-tagged Rif1 protein or its derivatives were expressed in 293T cells on the ver3-4 vector and purified as previously described^31,32^. The proteins were further purified with monoQ ion-exchange column or glycerol gradient centrifugation, if necessary.

### G4 DNA and Rif1BS DNA

The sequences of the oligonucleotides used in the assays are described in each figure. The oligonucleotides were OPC column-purified. Most of them were further purified by PAGE containing 8M urea. T_6_G_24_ and T_6_(GA)_12_ were always purified from urea-PAGE. The purity of the used oligonucleotides is shown in Supplementary Fig. S16. Oligonucleotides were heat-denatured at 96 °C for 3 min and gradually cooled down to room temperature in 50 mM KCl and 40% PEG200^34,58,59^.

### Expression and purification of fission yeast Taz1 protein and the substrate DNA

The Taz1 coding frame was amplified by RT-PCR from fission yeast cells by using the primer set (Taz1-N[Bam]: CGGGATCCATGATAAGCGTGCAAAGTACAGAAA and Taz1-C[Bam] CGGGATCCAGATTGATAATTAACAAGCTCTT) and was cloned into ver3-4 vector and N-terminally His_6_ and C-terminally FLAG_3_-tagged Taz1 was expressed in mammalian cells, and purified by anti-Flag affinity column followed by nickel column. The 21 bp ^32^P-end labeled double-stranded DNA containing Taz1 binding site (^32^P-TTACAGGTTACAGGTTACAGG/CCTGTAACCTGTAACCTGTAA) was generated by annealing of the two oligonucleotides, purified from PAGE, and used as a substrate for DNA binding assays.

### Pull-down assays with biotinylated DNA

5′-biotinylated T_6_G_24_ oligonucleotide was incubated in gel shift assay buffers (40 mM Hepes-KOH [pH 7.6], 50 mM KCl, 1 mM EDTA, 10% glycerol and 0.01% Triton X-100) in the presence or absence of Rif1 protein with ^32^P-labeled single-stranded DNA that Rif1 can bind to. Both biotinylated DNA and ^32^P-DNA had been heat-treated and denatured in KCl-PEG200 before used in the assays. After incubation for 30 min at room temperature, Dynabeads M-280 Streptavidin was added and the beads were extensively washed by the same buffer or that containing 1 M NaCl. Beads were resuspended in 80% formamide containing 1 mM EDTA, boiled for 3 min and run on 12% PAGE containing 8 M urea in 0.5x TBE.

### Analyses of DNA and protein-DNA complexes on polyacrylamide gels

Labeled DNA fragments or cold DNA fragments were mixed with purified proteins in reaction mixtures (10 µl or 20 µl) containing 40 mM Hepes-KOH (pH 7.6), 50 mM KCl, 1 mM EDTA, 10% glycerol, and 0.01% Triton X-100. After incubation at room temperature for 30 min, the reaction mixtures were directly applied onto a polyacrylamide gel. DNA and Protein-DNA complexes were analyzed on polyacrylamide gels prepared in 1x TBE, 50 mM KCl and 40% PEG200 or on those prepared in 1x TBE, 50 mM KCl (or other salt or no salt, where indicated) and 10% glycerol. Other gel electrophoresis conditions were also used, which are indicated in figure legends. For analyses of denatured DNA, DNA were heat-denatured in 95% formamide containing 5 mM EDTA (and LiCl where indicated) and were analyzed on PAGE containing 8 M urea in 0.5x TBE. The experiments were performed at least two times (in most cases three times or more) independently, and standard deviation and p-value by two-tailed student’s t-test were determined and presented, where indicated.

### Analytical gel-filtration analyses of Rif1 protein

Fifty µl of a peak fraction of the Ni^2+^-NTA column (0.22 µm-filtrated) was applied to pre-equilibrated Superose 6 PC3.2/30 column (GE Healthcare), and run in 20 mM Tris-HCl [pH7.8], 150 mM KCl, 1 mM EDTA, 0.5 mM DTT, 10% glycerol and protease inhibitors [cOmplete^™^ Protease Inhibitor Cocktail; Roche] at 40 µl/min at 4 °C. Eighty µl-fractions were collected and subjected to SDS-PAGE followed by western blotting with anti-DDDDK antibody (FLA-1, MBL Co.) to detect the C-terminal FLAG_3_ tag. Molecular size standards (GE Healthcare) were chromatographed under the same condition, and retention volumes of all the standards were determined by monitoring UV absorbance at 260 nm.

### Analytical glycerol-gradient centrifugation analyses of Rif1 protein

In 2.2-ml centrifuge tubes, 15–36% glycerol-gradient was made in 20 mM Tris-HCl (pH7.8), 150 mM KCl, 1 mM EDTA, 0.5 mM DTT and cOmplete^™^ Protease Inhibitor Cocktail (Roche). Twenty µl of a peak fraction of the Ni^2+^-NTA column (0.22 µm-filtrated) was diluted 5-fold, and layered on top of the gradient. One hundred µl of molecular size standards was similarly layered on top of another tube. These tubes were centrifuged at 40,000 rpm for 16 hr in Beckman TLS55 rotor at 4 °C. 0.1 ml-fractions were removed from top to bottom, and subjected to SDS-PAGE followed by anti-DDDDK blotting. The sedimentation pattern of molecular size standards was analyzed by SDS-PAGE followed by silver-staining.

### Analysis of hydrodynamic behaviors of Rif1 and its degradation products

Both *S*- and *Rs*-values of Rif1 and its degradation products were calculated from the data of glycerol-gradient centrifugation and gel-filtration after linear approximation to those of molecular size standards. Then, their native molecular weights were estimated as previously reported^44,45^.

### Fission yeast strains, medium and general techniques

All strains used in this study were previously described^6^. Methods for genetic and biochemical analyses of fission yeast have been described previously^60,61^. YES media containing 0.5% yeast extract, 3% glucose and 0.1 mg/ml each of adenine, uracil, leucine, lysine and histidine were used for cell culture and YES plates were made by adding 2% agar to YES media. 0.2 mg/ml G418 was added to YES medium for selection of kanMX. For 5-fluoroorotic acid (5-FOA) selection, 0.1 mg/ml 5-FOA was added to the media containing 6.3 g/L synthetic dextrose minimal medium (SD), 2% glucose and 0.1 mg/ml each of adenine, uracil and leucine.

### Circular Dichroism (CD) spectrometry

The oligonucleotides were diluted to 2 μM in 50 mM Tris-HCl (pH 7.5) without salt, or that with 50 mM KCl or that with 50 mM NaCl. Subsequently, these solutions were annealed by heating at 99 °C for 5 min, then slowly cooled to room temperature, and incubated overnight. Circular Dichroism (CD) spectra were recorded on a J-720 spectropolarimeter (JASCO, Tokyo, JAPAN) using a quartz cell (Agilent, microcell 50 μL, 10 mm optical path length) with scanning speed of 500 nm/min and a response time of 1 sec over a wavelength range of 230–320 nm. The CD spectra shown are representatives of five averaged scans taken at 25 °C or at various temperatures, as indicated in the figures. See Supplementary Figs S8,S9 for data and Supplementary Table S1 for summary.

## Supplementary information

Supplementary Information

## Data Availability

All data generated and analyzed in this study are included in this published article and its Supplementary Information.
