# Supplementary material for: Rif1 promotes association of G-quadruplex (G4) by its specific G4 binding and oligomerization activities
Source: Sci Rep. 2019 Jun 13;9:8618. doi: 10.1038/s41598-019-44736-9 (PMC6565636; doi:10.1038/s41598-019-44736-9)
Supplement: Supplementary file 1 — Supplementary Information [file 41598_2019_44736_MOESM1_ESM.pdf]

Supplementary Information for

Title:

Rif1 promotes association of G-quadruplex (G4) by its specific G4 binding and oligomerization activities

Authors:
***Hisao Masai, Rino Fukatsu, Naoko Kakusho, Yutaka Kanoh, Kenji Moriyama, Yue Ma, Keisuke Iida, Kazuo Nagasawa**

Correspondence should be addressed to
Hisao Masai
E-mail: masai-hs@igkakuken.or.jp

Contents

Legends to Supplementary Figures
Supplementary Table S1
Supplementary Figures (Supplementary Figures S1-S16)

Legends to Supplementary Figures

Supplementary Figure S1.
Comparison of DNA binding activities of the full-length and 70 kDa C-terminal polypeptide of Rif1.

A. Protein fractions containing the full-length or 70 kDa polypeptide of Rif1 were analyzed on 5-20% gradient SDS-PAGE and proteins were stained by silver. Lane 1, full-length Rif1 protein purified by consecutive steps of anti-FLAG affinity column, nickel column and monoQ column chromatography (a different fraction from that used in Fig. 1). Lane 2, the 70 kDa polypeptide fraction purified by glycerol gradient centrifugation of FLAG-column purified material. Lane 3, the 70 kDa polypeptide fraction purified by monoQ column chromatography of the material enriched by consecutive steps of anti-FLAG affinity column and nickel column. Closed and open triangles indicate the full-length and the 70 kDa C-terminal polypeptide of Rif1, respectively. In the fractions for lanes 2 and 3, the full-length Rif1 was not detected even with western blotting (not shown). The protein concentrations were estimated by comparison with the BSA marker that was run in the same gel (not shown). Lane 1 and lanes 2/3 were from different gels; therefore, the band intensities cannot be directly compared. Original full-length gels are shown in Supplementary Fig. S17. **B**. The end-labeled T_6G_{24} DNA (0.25 pmole), which had been heat-denatured and renatured in 50 mM KCl and 40% PEG200, were incubated with increasing amounts of Rif1 protein, as shown, and were analyzed on 8% PAGE containing 50 mM KCl and 40% PEG200 in 1x TBE. **C**. Band intensities of the gel were measured and relative binding efficiency was estimated as described in the legend to Fig. 2. The concentrations of the protein on the X-axis are based on those of the full-length polypeptide alone. However, the preparation used contains four-fold molar excess of the C-terminal 70 kDa polypeptide. If this polypeptide is capable of binding to G4 and is also taken into account, the concentrations of the polypeptides could be as much as five-times larger. Even that is the case, the Kd would be ~1.5 nM (assuming that the 70 kDa polypeptide binds to the G4 substrate as efficiently as the full-length). However, the former protein binds to G4 with much less affinity than the latter does. Thus, the full-length Rif1 protein mediates high-affinity binding to G4. Molecular weight marker is ϕX174 DNA digested by *Hae*III.

Supplementary Figure 2 (related to Fig. 2).
Binding of Rif1 to various single-stranded DNAs derived from sequences known to form specific G4 configuration.

Single-stranded oligonucleotide DNAs (0.25 pmole) as shown, which had been heat-denatured and renatured in 50 mM KCl and 40% PEG200, were incubated in the presence of increasing amounts of Rif1 protein (0, 0.3, 0.75, and 1.5 nM) and were analyzed on 12% PAGE (29:1; 1x TBE, 40% PEG200 and 50 mM KCl). The sequences of the oligonucleotides are shown in Fig. 2. The graph shows quantification of the Rif1 binding to each oligonucleotide. The values were calculated by dividing the radioactivity of the shifted bands (complex) by the sum of the free G4-structured and the shifted bands. The ticks represent the sizes of 310, 271/281, 234, 194, 118 and 72 bp, from the top. The estimated Kd values are also shown in the table. ND: not determined.

Supplementary Figure S3 (related to Fig. 3).
Binding of Rif1 to various single-stranded DNAs and their derivatives.
The single-stranded oligonucleotide DNAs (0.25 pmole) as shown, which had been heat-denatured and renatured in 50 mM KCl and 40 % PEG200, were incubated in the absence (-) or presence (+) of Rif1 protein (10 fmole [1nM] of the Rif1 full-length polypeptide; the preparation used contains ~10-fold molar excess of degradation products), and were analyzed on 12% PAGE (1x TBE, 50 mM KCl and 40 % PEG200). List of oligonucleotides used in the assays and their sequences are shown in Fig. 3.

Supplementary Figure S4 (related to Fig. 4).
Binding of Rif1 to various single-stranded DNAs and their derivatives: stimulation of Rif1 binding by runs of guanine at the 3'-end of the DNA (another one of the triplicate experiments shown in Fig. 4)
The results of an independent set of assay identical to Fig. 4 are shown.

Supplementary Figure S5
Analyses of oligonucleotide used in this study on polyacrylamide gel.
Oligonucleotides shown (0.25 pmole), denatured and reannealed in 50 mM KCl and 40% PEG200, were run on 10% PAGE (29:1; 1xTBE, 50 mM KCl and 10% PEG200), and autoradiographed. The oligonucleotides in red, pink, and black represent good, fair and poor binders of Rif1, respectively. The bands indicated by pink bars show the slow migrating forms. T95_2T_GGG, that shows mobility-shifter forms (indicated by a dotted pink line), is a weak but better binder than T95_2T_G and T95_2T_GG, which show little slow-migrating forms.

Supplementary Figure S6.
Binding of Rif1 to small oligonucleotides derived from Rif1BS consensus sequence: evaluation of AGTG$_n$ sequences and effect of a mutation at the conserved AGT sequence.
The single-stranded oligonucleotide DNAs (0.1 pmole) as shown, which had been heat-denatured and renatured in 50 mM KCl and 40% PEG200, were incubated in the presence (+, 25 fmole [2.5 nM] of the Rif1 full-length polypeptide; the preparation used contains ~10-fold molar excess of degradation products as well) or absence (-) of Rif1 protein. Samples were analyzed on 14% (**A** [left]) or 12% (**A** [right] and **B**) PAGE (1x TBE, 50 mM KCl and 40% PEG200). The binding assays were conducted in separate experiments two times with similar results, and only the representative data are presented. The graph shows quantification of the Rif1 binding to each oligonucleotide. ds: 12 bp double-stranded DNA (CGCGAATTCGCG). M: molecular weight marker (ϕX174 DNA digested by *Hae*III). The ticks represent the sizes of 310, 271/281, 234, 194, 118 and 72 bp, from the top.

Supplementary Figure S7.
Binding of Rif1 to small G-tract oligonucleotides.
The single-stranded oligonucleotide DNAs (0.25 pmole) as shown, which had been heat-denatured and renatured in 50 mM KCl and 40% PEG200, were incubated with increasing amounts of the Rif1 full-length polypeptide, as shown in the figure. Samples

were analyzed on 12% PAGE (1x TBE, 50 mM KCl and 40% PEG200). The ticks represent the sizes of 600, 310, 271/281, 234, 194, 118 and 72 bp, from the top.

Supplementary Figure S8.
CD patterns of the oligonucleotides analyzed in this study.
CD measurement was conducted at 2µM DNA in 50 mM Tris-Cl (pH 7.5) without salt (black) or in 50 mM Tris-Cl (pH 7.5) containing 50 mM KCl (red) or 50 mM NaCl (blue). The sequence and relative binding affinity to Rif1 in the presence of KCl are also indicated for each oligonucleotide (-, +, ++, +++). The criteria of binding affinity is defined in the legend to Fig. 3. The topology of DNA estimated form the CD pattern is also presented in red (50 mM KCl) or in blue (50 mM NaCl). CD of Rif1-8, derived from $Rif1BS_{I:4255}$, was measured only in the presence of 100 mM KCl. Summary of this measurement is shown in **Supplementary Table S1**.

Supplementary Figure S9.
Determination of melting temperatures of selected G4-forming sequences used in this study.
CD profiles were measured at various temperatures for a selected set of G4-forming sequences (with 50 mM and 10 mM KCl), as described in the legend to Supplementary Fig. S7. Tm for each sequence was estimated from the melting curve. CEB1 and CEB1_TA are capable of maintaining the parallel form even at 95°C, and thus their Tm are probably higher than 95°C.

Supplementary Figure S10 (related to Fig. 5).
Effect of salt on Rif1 binding to selected G4 oligonucleotides whose topologies change in response to salt (another one of the triplicate experiments shown in Fig. 5).
The results of an independent set of assays identical to Fig. 5 are shown.

Supplementary Figure S11.
Calculation of native molecular weights of the full-length Rif1 and the ~70kDa degraded polypeptide.
The values for both *S* (sedimentation coefficient; *left-most graph and table*) and *Rs* (Stokes radius) were determined. *Rs* was determined according either to Siegel and Monty (*middle graph and middle table*; ref. 44) or to Erickson (*right graph and right table*; ref 45). The native molecular weight (Native Mw.) and the oligomeric state (Multimer), calculated with the equation below the table (left side), are presented in the middle and right tables. *Smax/S* values were also calculated and are presented in the same tables. General interpretation of *Smax/S*, indicative of molecular shape, is given in a box below the tables.

Supplementary Figure S12.
Effect of Taz1 on interaction of Rif1 with DNA
A. Purification of fission yeast Taz1 protein (663 aa). His_6-Rif1-$Flag_3$ protein expressed in human embryonic kidney 293T cells was purified by consecutive anti-Flag and nickel columns. Eluates from nickel column were analyzed on SDS-PAGE (5-20% gradient gel) and stained by silver. **B**. ^{32}P-end labeled Htelo3 DNA (0.25 pmole) was mixed with

the combination of Taz1 and Rif1 proteins, as indicated, and was analyzed on 8% PAGE (29:1; 1x TBE, 40% 50 mM KCl and 1 PEG200). **C**. and **D**. ^{32}P-end labeled Taz1BS dsDNA (0.25 pmole) was mixed with the combination of Taz1 and Rif1 proteins, as indicated, and was analyzed on 6% PAGE (29:1; 1xTBE and 10% glycerol) (C) or 8%PAGE (29:1; 1xTBE, 50 mM KCl, and 40% PEG200) (D). Ticks in the ϕX174/ HaeIII markers (B) represent 600, 310, 271/281, 234, 194, 118 and 72 bp.

Supplementary Figure S13.
Ladder formation in denaturing PAGE.
A.^{32}P-labeled Rif1-8 (0.25 pmole), denatured and reannealed in 100 mM KCl and 40% PEG200, were run on 6 % PAGE (29:1) with 8M urea in 0.5x TBE, and autoradiographed. **B**. ^{32}P-labeled T_6G_{24} (0.25 pmole), denatured and reannealed in 100 mM KCl and 40% PEG200, were run on 8 % PAGE (29:1) with 8M urea in 0.5x TBE, and autoradiographed. In both A and B, DNAs were pretreated with different concentration of formamide in the absence (lanes 1-6) or presence (lanes 7-12) of 1M NaCl. DNAs were heated at 96°C for 1 min before loading onto PAGE (lanes 1-5, 7-11). Ladders are still generated in a denaturing gel after heat denaturation in formamide, suggesting that the structures may not be simply stacked oligomers, but could include intertwined G4 structures made of multiple DNA strands.

Supplementary Figure S14.
The length of G-tracts present in Rif1BS.
The sequences of the 1 kb segments encompassing the 35 Rif1BS were analyzed. All the G-tracts equal or longer than 3 were extracted, and the numbers of 3G~7G were scored for the “top 10” and “bottom 10” Rif1BS. The analyses were conducted for both strands. The G-strand represents the strand on which the G-tracts of Rif1CS are present, and the C-strand the other strand. Fractions of each G-tract are shown.

Supplementary Figure S15.
Frequency of various dinucleotide sequences preceding the G-tracts (n=3 or longer) in Rif1BS.
The sequences of the 1 kb segments encompassing the 35 Rif1BS were analyzed. All the G-tracts equal or longer than 3 were extracted, and the preceding dinucleotides were scored. The analysis was conducted on both strands. The G-strand represents the strand on which the G-tracts of Rif1CS are present, and the C-strand the other strand. Fractions of each dinucleotide are shown.

Supplementary Figure S16.
Analyses of labeled oligonucleotides on denaturing PAGE containing 8M urea.
Oligonucleotides used in this studies were purified by gel filtration, end-labeled by T4 polynucleotide kinase, and denatured in 95% formamide containing 5 mM EDTA and 20 mM LiCl, and were run on 15% PAGE containing 8M urea in 0.5x TBE. T_6G_{24} appears as a ladder, since it forms secondary structures even in the presence of 8M urea. The bands at the bottom of the gels are free labeled nucleotides that remained after gel filtration purification of labeled oligonucleotides. In lane11, the oligonucleotide (12mer) was too small to be separated on this gel and ran off the gel.

Supplementary Figure S17.
Original full-length gels for data in Supplementary Fig. S1.
Figure S1A lane 1: gel1
Figure S1A lane 2: gel2
Figure S1A lane 3: gel3

Supplementary Figure S18.
List of the selected oligonucleotides used in this study and their reported structures.

Name	Sequence	Topology (reported)	Topology in KCl (observed)	Topology in NaCl (observed)	Rif1 binding	Rif1 binding in NaCl	Rif1 binding without salt
Pu24T	TGAGGGTGGTGAGGGTGGGGA	Parallel	Parallel	Non-G4?	++		
CEB25	AAGGGTGGGTGTAAGTGTGGGTGGG T	Parallel	Parallel	Parallel?	+		
HT	TTGGGTTAGGGTTAGGGTTAGGGA	Hybrid (form1)	Hybrid	Anti-Parallel	-		
Htelo1	TAGGGTTAGGGTTAGGGTTAGGG	Hybrid (form1)	Hybrid	Anti-Parallel	-		
Htelo2	TAGGGTTAGGGTTAGGGTTAGGGTT	Hybrid (form2)	Hybrid	Anti-Parallel	-		
Htelo3	GGGTTAGGGTTAGGGTTAGGGT	Anti-Parallel	Hybrid	Anti-Parallel	-		
DX1 (duplex)	CGCGAATTCGCG		Non-G4	Non-G4	-		
DX2 (hairpin)	ATCTGAGAATCAGAT		Non-G4	Non-G4	-		
TERC18 (RNA)	GGGUUGCGGAGGGUGGGC	Parallel (dimeric)	Parallel	Parallel?	+		
T_6G_{24}	TTTTTTGGGGGGGGGGGGGGGGGGG GGGG	a propeller-type parallel	Parallel	Parallel	+++	++	+
Htelo4	AGGGCTAGGGCTAGGGCTAGGG	Chair	Anti-Parallel	Anti-Parallel	-		
Htelo4_3nt_spacer	AGGGCTAGGGCTACTAGGGCTAGGG		Anti-Parallel	Anti-Parallel	-		
Htelo4(GGGGGG)	AGGGCTAGGGCTAGGGGGG		Hybrid or Anti-Parallel	Mix or Anti-Parallel	++		
Htelo4(GGGGGG)_3nt_spacer	AGGGCTAGGGCTACTAGGGGGG		Hybrid?	Anti-Parallel	++	+	+
$T_6(GA)_{12}$	TTTTTTGAGAGAGAGAGAGAGAGAG AGAGA		Non-G4	Non-G4	-		
CEB1	AGGGGGGAGGGAGGGTGG	Parallel (dimeric)	Parallel	Parallel?	++		
CEB1_A	AGGGAGGGAGGGAGGGTGG		Parallel	Parallel?	++		
CEB1_TA	AGGGTAGGGAGGGAGGGTGG		Parallel	Non-G4?	++	-	-
CEB1_TTA	AGGGTTAGGGAGGGAGGGTGG		Parallel	Non-G4	++	-	-
CEB1_ΔGG	AGGGGGGAGGGAGGGT		Parallel	Parallel?	++		
Htelo1	TAGGGTTAGGGTTAGGGTTAGGG		Hybrid	Anti-Parallel	-		
Htelo1_A	TAGGGAGGGTTAGGGTTAGGG		Hybrid or Mix	Anti-Parallel or Mix	-		
Htelo1_no_spacer	TAGGGGGGTTAGGGTTAGGG		Parallel	Anti-Parallel or Mix	++		
Htelo1_no_spacer_2	TAGGGTTAGGGTTAGGGGGG		Mix?	Anti-Parallel or Mix	+++	+	+
93del	GGGGTGGGAGGAGGGT	Parallel (dimeric)	Parallel	Parallel	++		
93del_ΔG	GGGTGGGAGGAGGGT		Parallel	Parallel	+		
T95_2T	TTGGGTGGGTGGGTGGGT	Parallel	Parallel	Parallel	+		
T95_2T_G	TTGGGGTGGGTGGGTGGGT		Parallel	Parallel	-		
T95_2T_GG	TTGGGGGTGGGTGGGTGGGT		Parallel	Parallel	-		
T95_2T_GGG	TTGGGGGGTGGGTGGGTGGGT		Parallel	Parallel	-		
Rif1-8	GTGGGGGATGTGGGATAGACTATTTT AGACTTGAGACTGTGTATTCACTTAA AAGTGCTATGTGGGGGCA		Parallel		++		

Supplementary Table S1 List of G4-related oligonucleotides used in this study: sequence, topology and Rif1 binding

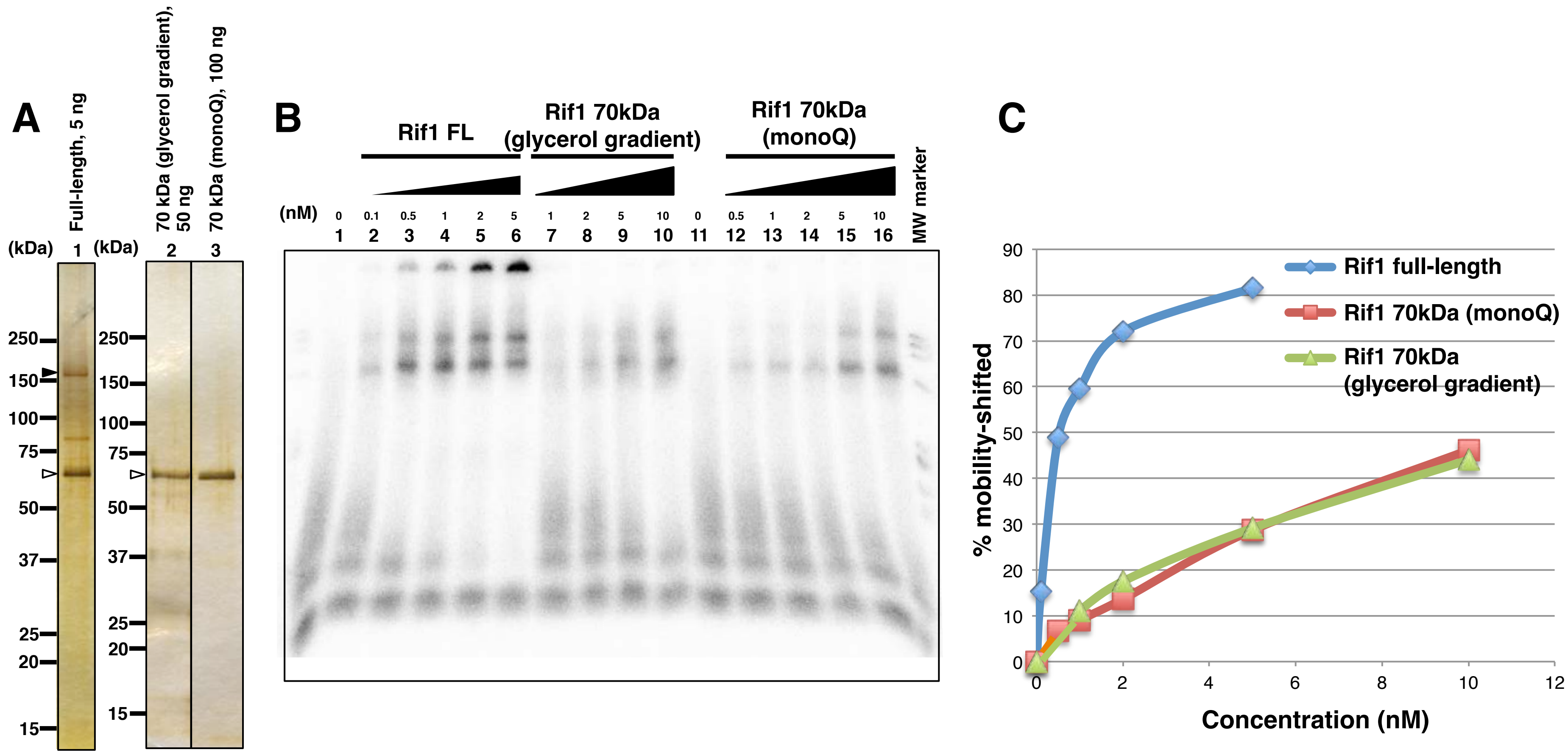

Supplementary Figure S1.

Comparison of DNA binding activities of the full-length and 70 kDa C-terminal polypeptide of Rif1.

A. Protein fractions containing the full-length or 70 kDa polypeptide of Rif1 were analyzed on 5-20% gradient SDS-PAGE and proteins were stained by silver. Lane 1, full-length Rif1 protein purified by consecutive steps of anti-FLAG affinity column, nickel column and monoQ column chromatography (a different fraction from that used in Fig. 1). Lane 2, the 70 kDa polypeptide fraction purified by glycerol gradient centrifugation of the FLAG-column purified material. Lane 3, the 70 kDa polypeptide fraction purified by monoQ column chromatography of the material enriched by consecutive steps of anti-FLAG affinity column and nickel column. Closed and open triangles indicate the full-length and the 70 kDa C-terminal polypeptide of Rif1, respectively. In the fractions for lanes 2 and 3, the full-length Rif1 was not detected even with western blotting (not shown). The protein concentrations were estimated by comparison with the BSA marker that was run in the same gel (not shown). Lane 1 and lanes 2/3 were from different gels; therefore, the band intensities cannot be directly compared. Original full-length gels are shown in Supplementary Fig. S17. **B**. The end-labeled T_6G_{24} DNA (0.25 pmole), which had been heat-denatured and renatured in 50 mM KCl and 40% PEG200, were incubated with increasing amounts of Rif1 protein, as shown, and were analyzed on 8% PAGE containing 50 mM KCl and 40% PEG200 in 1x TBE. **C**. Band intensities of the gel were measured and relative binding efficiency was estimated as described in the legend to Fig. 2. The concentrations of the protein on the X-axis are based on those of the full-length polypeptide alone. However, the preparation used contains four-fold molar excess of the C-terminal 70 kDa polypeptide. If this polypeptide is capable of binding to G4 and is also taken into account, the concentrations of the polypeptides could be as much as five-times larger. Even that is the case, the Kd would be ~1.5 nM (assuming that the 70 kDa polypeptide binds to the G4 substrate as efficiently as the full-length). However, the former protein binds to G4 with much less affinity than the latter does. Thus, the full-length Rif1 protein would mediate high-affinity binding to G4. Molecular weight marker is ϕX174 DNA digested by *Hae*III.

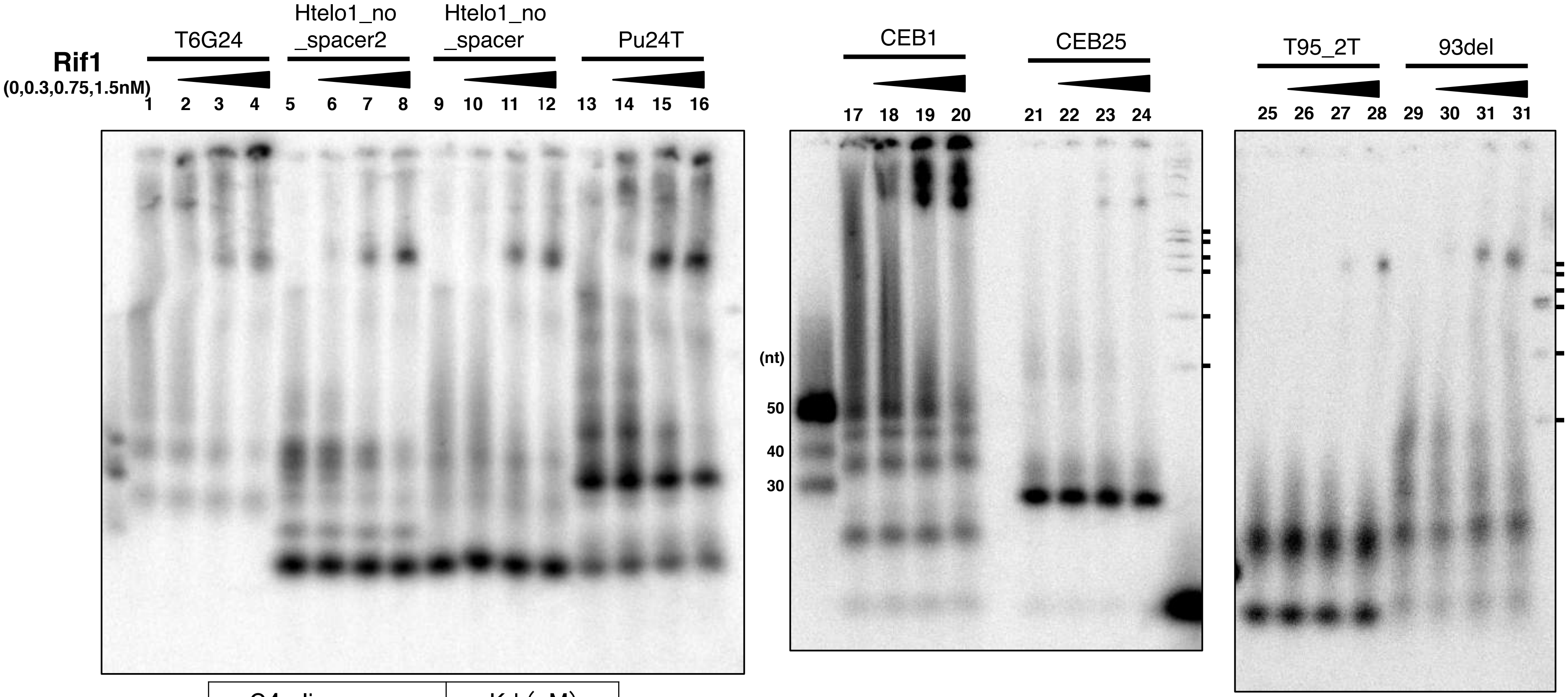

G4 oligo name	Kd (μM)
93del	ND
T95_2T	ND
CEB25	ND
CEB1	0.5
Pu24T	0.7
Htelo1_no_spacer	0.8
Htelo1_no_spacer2	0.9
T6G24	0.3

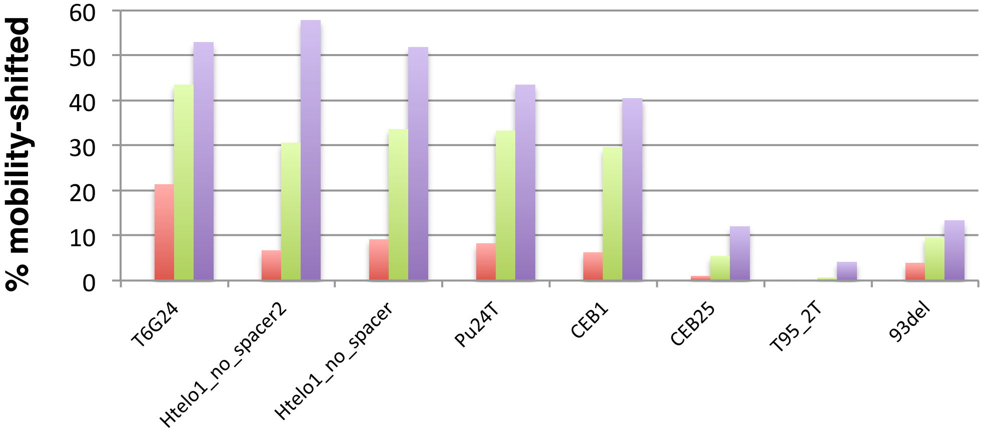

Supplementary Figure 2 (related to Fig. 2).
Binding of Rif1 to various single-stranded DNAs derived from sequences known to form specific G4 configuration.
Single-stranded oligonucleotide DNAs (0.25 pmole) as shown, which had been heat-denatured and renatured in 50 mM KCl and 40% PEG200, were incubated in the presence of increasing amounts of Rif1 protein (0, 0.3, 0.75, and 1.5 nM) and were analyzed on 12% PAGE (29:1; 1x TBE, 40% PEG200 and 50 mM KCl). The sequences of the oligonucleotides are shown in Fig. 2. The graph shows quantification of the Rif1 binding to each oligonucleotide. The values were calculated by dividing the radioactivity of the shifted bands (complex) by the sum of the free G4-structured and the shifted bands. The ticks represent the sizes of 310, 271/281, 234, 194, 118 and 72 bp, from the top. The estimated Kd values are also shown in the table. ND: not determined.

Masai *et al.* Supplementary Figure S3

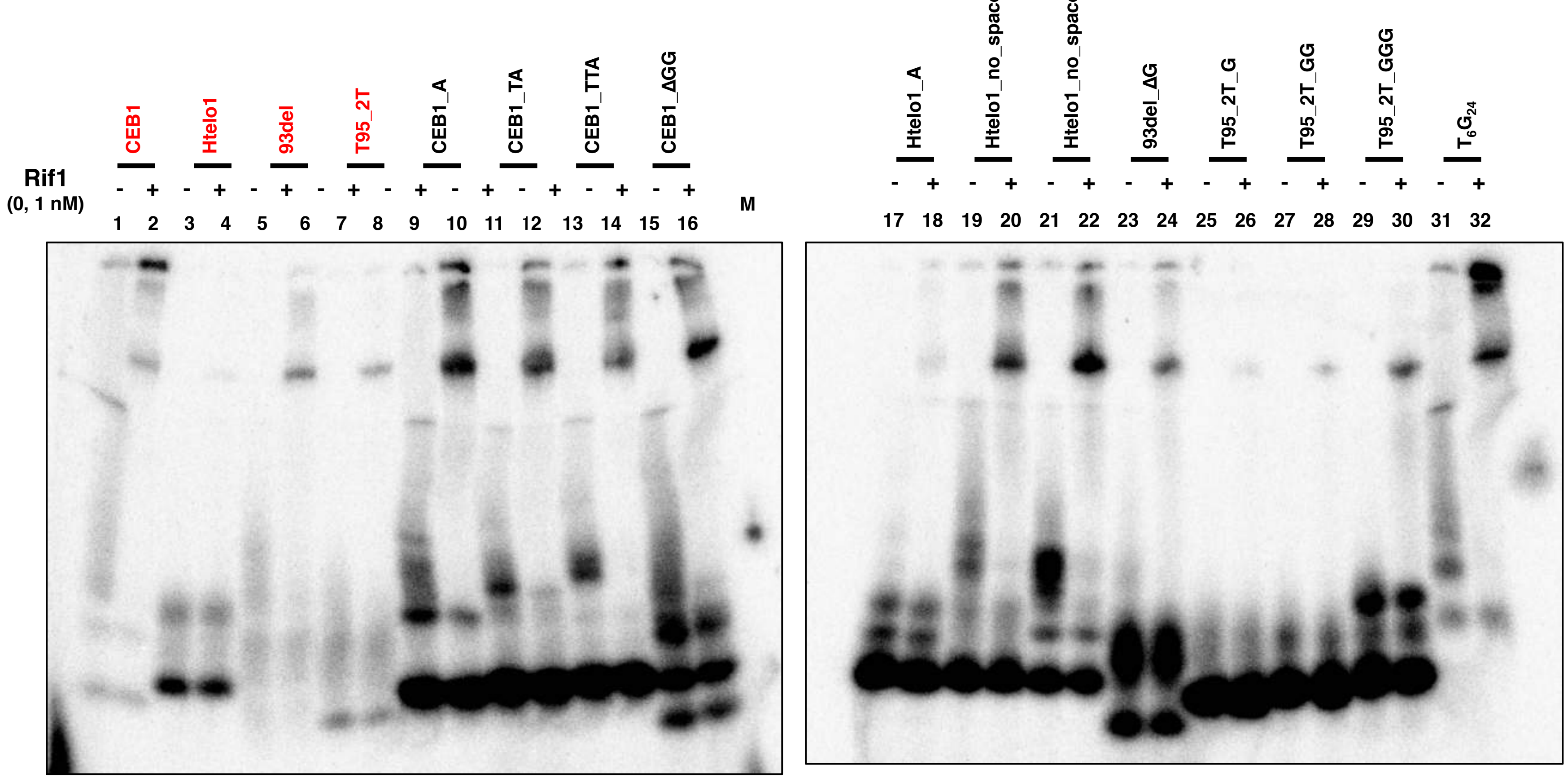

Supplementary Figure S3 (related to Fig. 3).

Binding of Rif1 to various single-stranded DNAs and their derivatives: effect of spacer sequence and runs of guanine on binding of Rif1.

The single-stranded oligonucleotide DNAs (0.25 pmole) as shown, which had been heat-denatured and renatured in 50 mM KCl and 40 % PEG200, were incubated in the absence (-) or presence (+) of Rif1 protein (10 fmole [1nM] of the Rif1 full-length polypeptide; the preparation used contains ~10-fold molar excess of degradation products), and were analyzed on 12% PAGE (1x TBE, 50 mM KCl and 40 % PEG200). List of oligonucleotides used in the assays and their sequences are shown in Fig. 3.

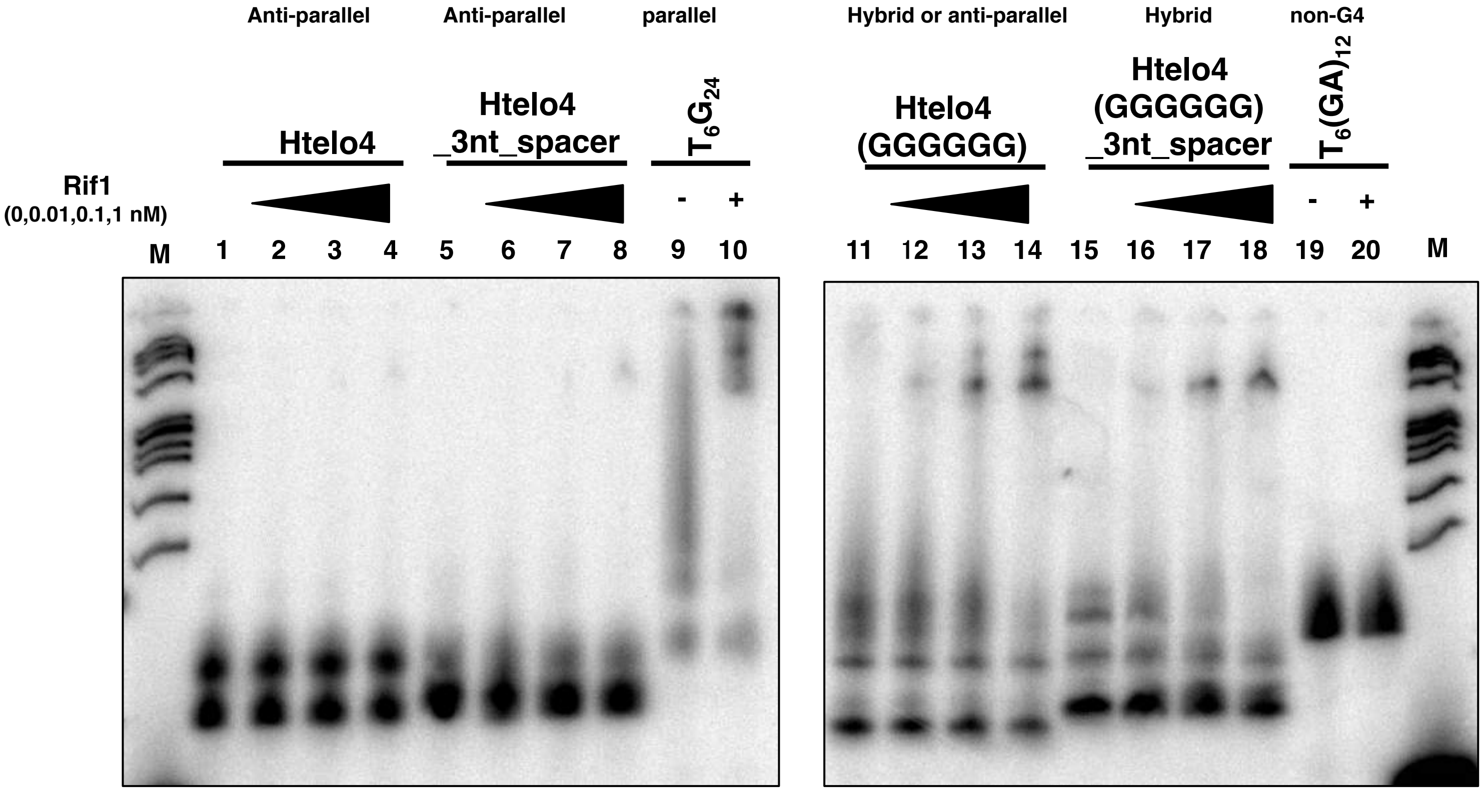

Supplementary Figure S4 (related to Fig. 4).
Binding of Rif1 to various single-stranded DNAs and their derivatives: stimulation of Rif1 binding by runs of guanine at the 3'-end of the DNA (another one of the triplicate experiments shown in Fig. 4).
The results of an independent set of assays identical to Fig. 4 are shown.

Htelo4: AGGGCTAGGGCTAGGGCTAGGG
Htelo4_3nt_spacer: AGGGCTAGGGCTACTAGGGCTAGGG
Htelo4(GGGGGG): AGGGCTAGGGCTAGGGGGG
Htelo4(GGGGGG)_3nt_spacer: AGGGCTAGGGCTACTAGGGGGG
T_6G_{24}: TTTTTTGGGGGGGGGGGGGGGGGGGGGGGG
$T_6(GA)_{12}$: TTTTTTGAGAGAGAGAGAGAGAGAGAGA

Masai *et al*. Supplementary Figure S5

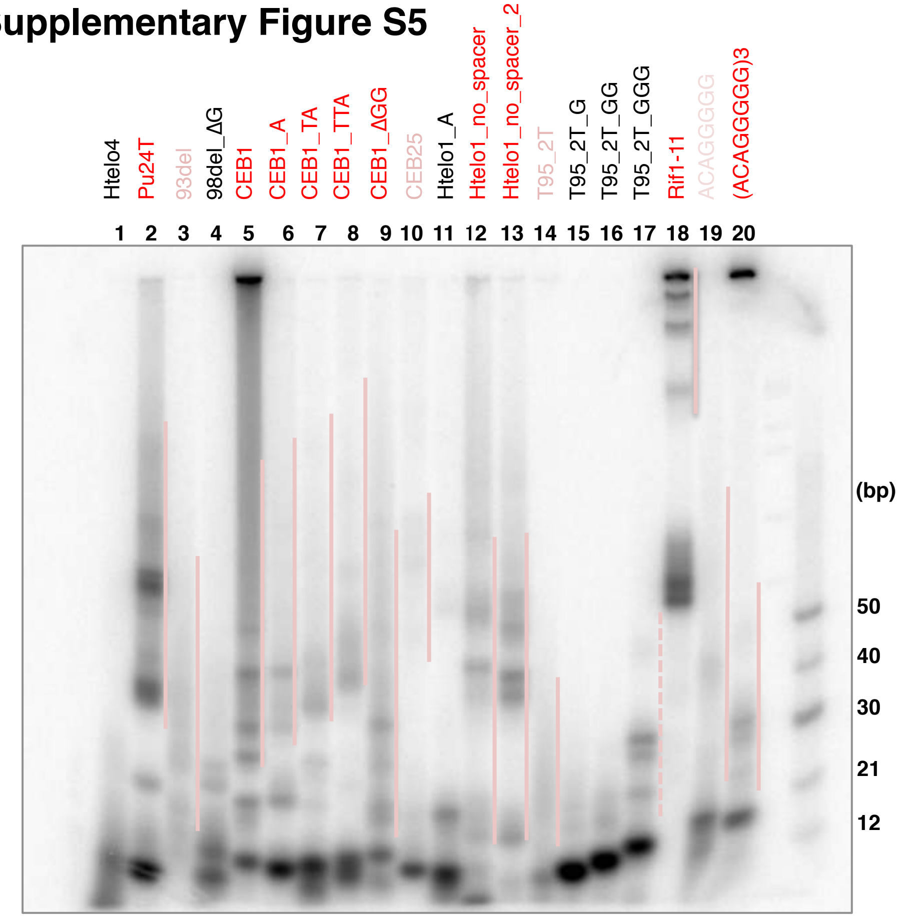

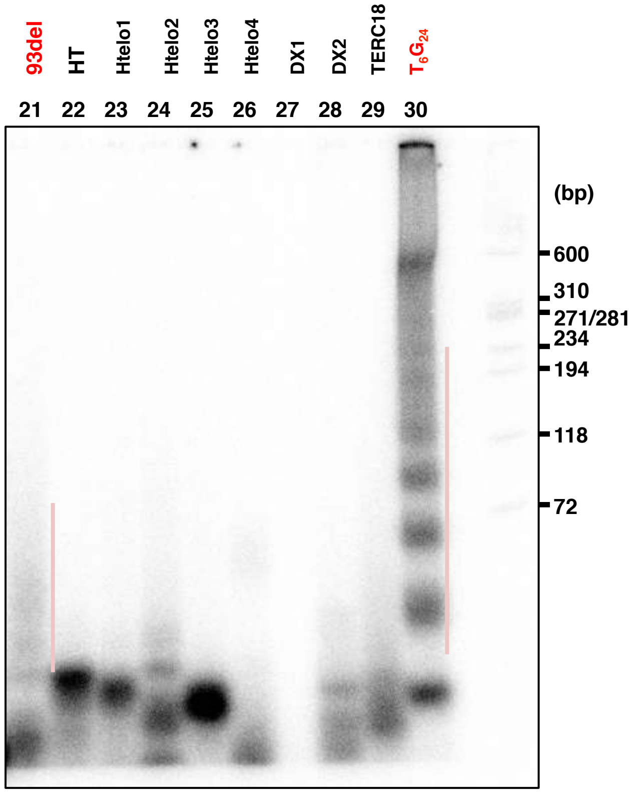

Htelo4:AGGGCTAGGGCTAGGGCTAGGG
Pu24T:TGAGGGTGGTGAGGGTGGGGA
93del:GGGGTGGGAGGAGGGT
93del_ΔG:GGGTGGGAGGAGGGT
CEB1:AGGGGGGAGGGAGGGTGG
CEB1_A:AGGGAGGGAGGGAGGGTGG
CEB1_TA:AGGGTAGGGAGGGAGGGTGG
CEB1_TTA:AGGGTTAGGGAGGGAGGGTGG
CEB1_ΔGG:AGGGGGGAGGGAGGGT
CEB25:AAGGGTGGGTGTAAGTGTGGGTGGGT
Htelo1:TAGGGTTAGGGTTAGGGTTAGGG
Htelo1_A:TAGGGAGGGTTAGGGTTAGGG
Htelo1_no_space:TAGGGGGGTTAGGGTTAGGG
Htelo1_no_spacer_2:TAGGGTTAGGGTTAGGGGGG
T95_2T:TTGGGTGGGTGGGTGGGT
T95_2T-G:TTGGGGTGGGTGGGTGGGT
T95_2T-GG:TTGGGGGTGGGTGGGTGGGT
T95_2T-GGG:TTGGGGGGTGGGTGGGTGGGT
Rif1-11:TTTGGGTCTA AGTGGGGGAT GTGGGATAGA CTATTTTAGA CTTGAGACTG
TGTATTCACT TAAAAGTGCT ATGTGGGGGC A

93del:GGGGTGGGAGGAGGGT
HT:TTGGGTTAGGGTTAGGGTTAGGGA
Htelo1:TAGGGTTAGGGTTAGGGTTAGGG
Htelo2:TAGGGTTAGGGTTAGGGTTAGGGTT
Htelo3:GGGTTAGGGTTAGGGTTAGGGT
Htelo4:AGGGCTAGGGCTAGGGCTAGGG
DX1:CGCGAATTCGCG
DX2:ATCTGAGAATCAGAT
TERC18:GGGUUGCGGAGGGUGGGC
T_6G_{24}:TTTTTTGGGGGGGGGGGGGGGGGGGGGGGG

Supplementary Figure S5
Analyses of oligonucleotide used in this study on polyacrylamide gel.
Oligonucleotides shown (0.25 pmole), denatured and reannealed in 50 mM KCl and 40% PEG200, were run on 10% PAGE (29:1; 1xTBE, 50 mM KCl and 10% PEG200), and autoradiographed. The oligonucleotides in red, pink, and black represent good, fair and poor binders of Rif1, respectively. The bands indicated by pink bars show the slow migrating forms.

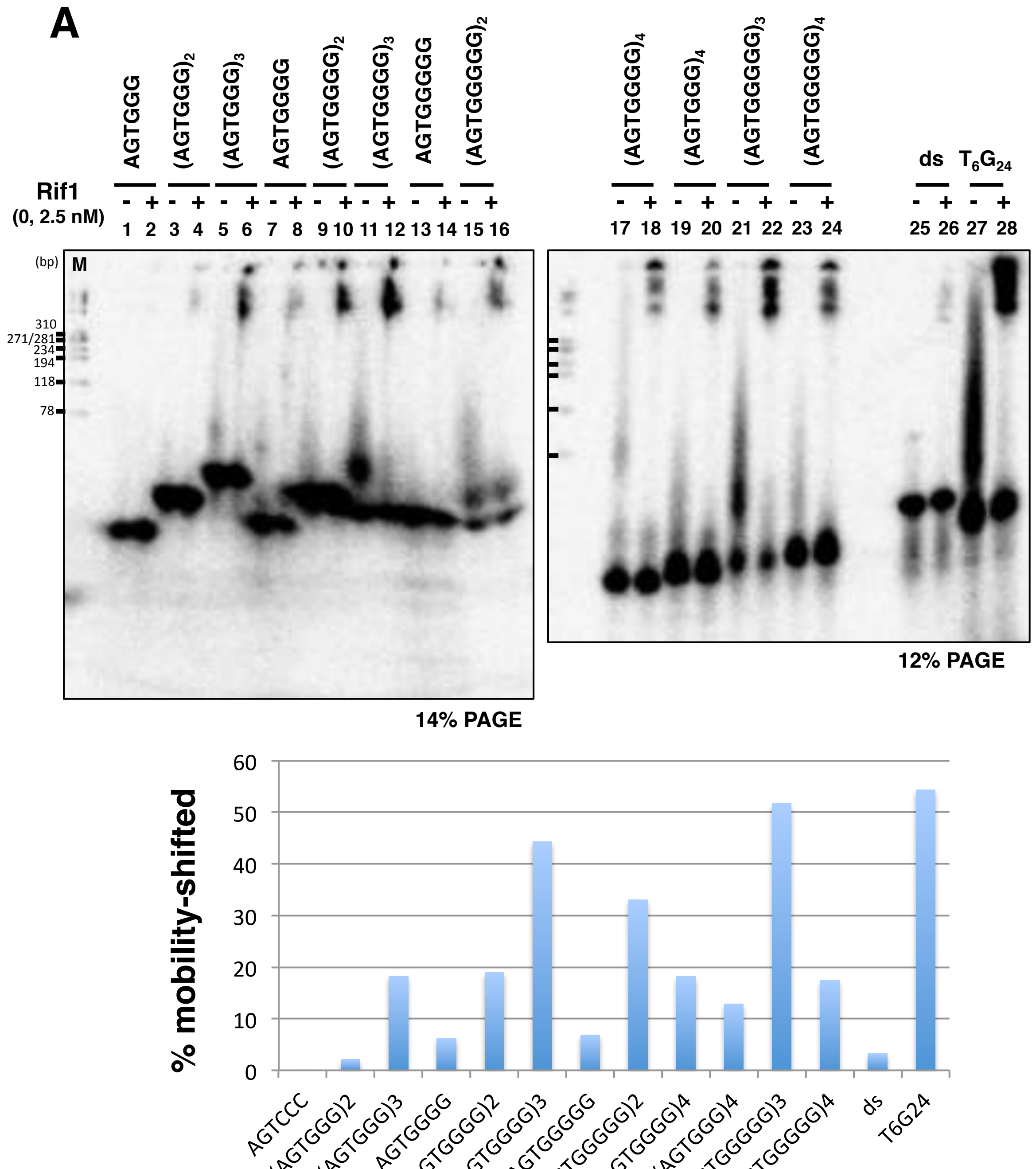

Masai *et al*. Supplementary Figure S6

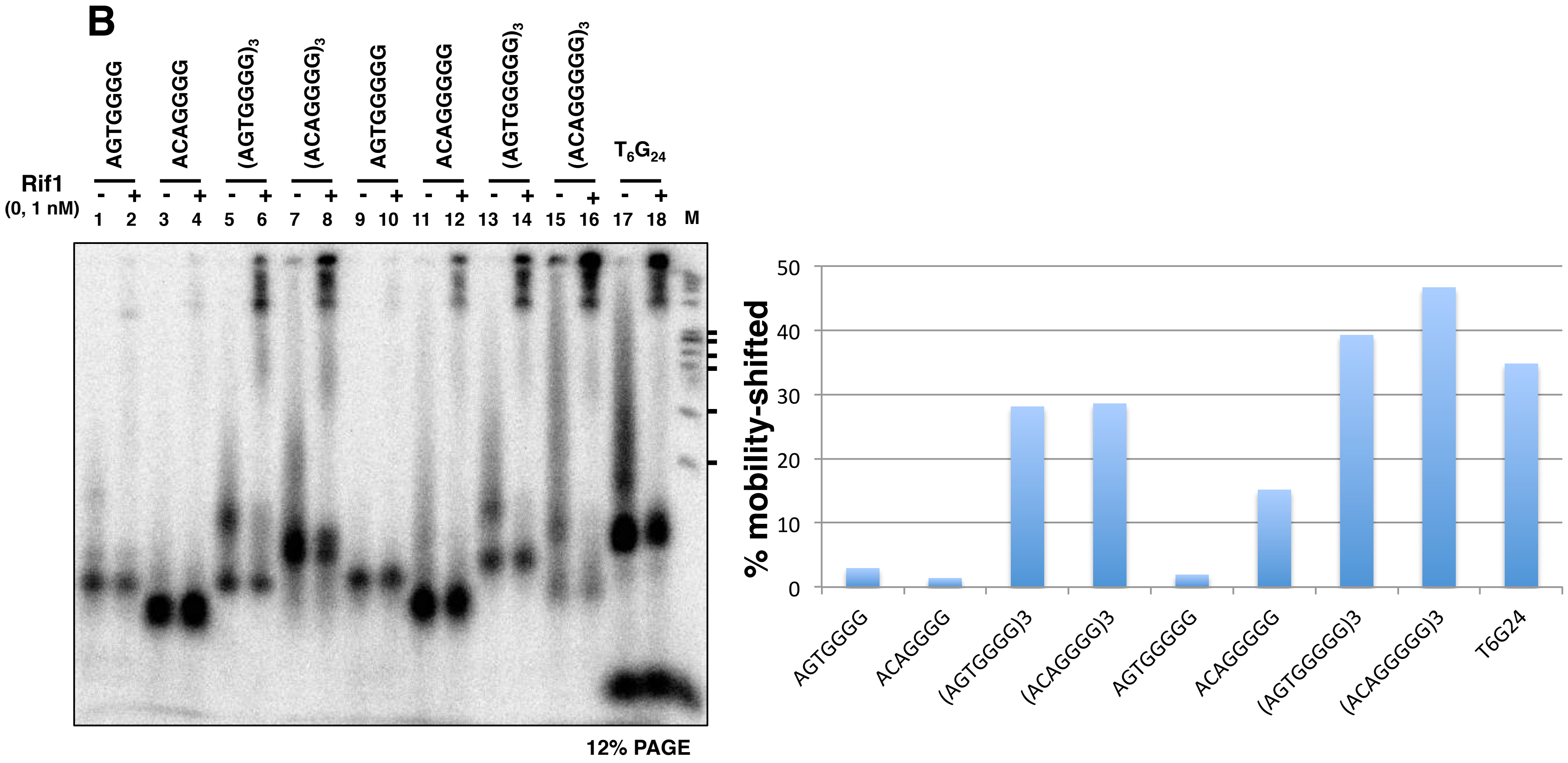

Supplementary Figure S6.

Binding of Rif1 to small oligonucleotides derived from Rif1BS consensus sequence: evaluation of AGTG$_n$ sequences and effect of a mutation at the conserved AGT sequence.

The single-stranded oligonucleotide DNAs (0.1 pmole) as shown, which had been heat-denatured and renatured in 50 mM KCl and 40% PEG200, were incubated in the presence (+, 25 fmole [2.5 nM] of the Rif1 full-length polypeptide; the preparation used contains ~10-fold molar excess of degradation products as well) or absence (-) of Rif1 protein. Samples were analyzed on 14% (**A** [left]) or 12% (**A** [right] and **B**) PAGE (1x TBE, 50 mM KCl and 40% PEG200). The binding assays were conducted in separate experiments two times with similar results, and only the representative data are presented. The graph shows quantification of the Rif1 binding to each oligonucleotide. ds: 12 bp double-stranded DNA (CGCGAATTCGCG). M: molecular weight marker (ϕX174 DNA digested by *Hae*III). The ticks represent the sizes of 310, 271/281, 234, 194, 118 and 72 bp, from the top.

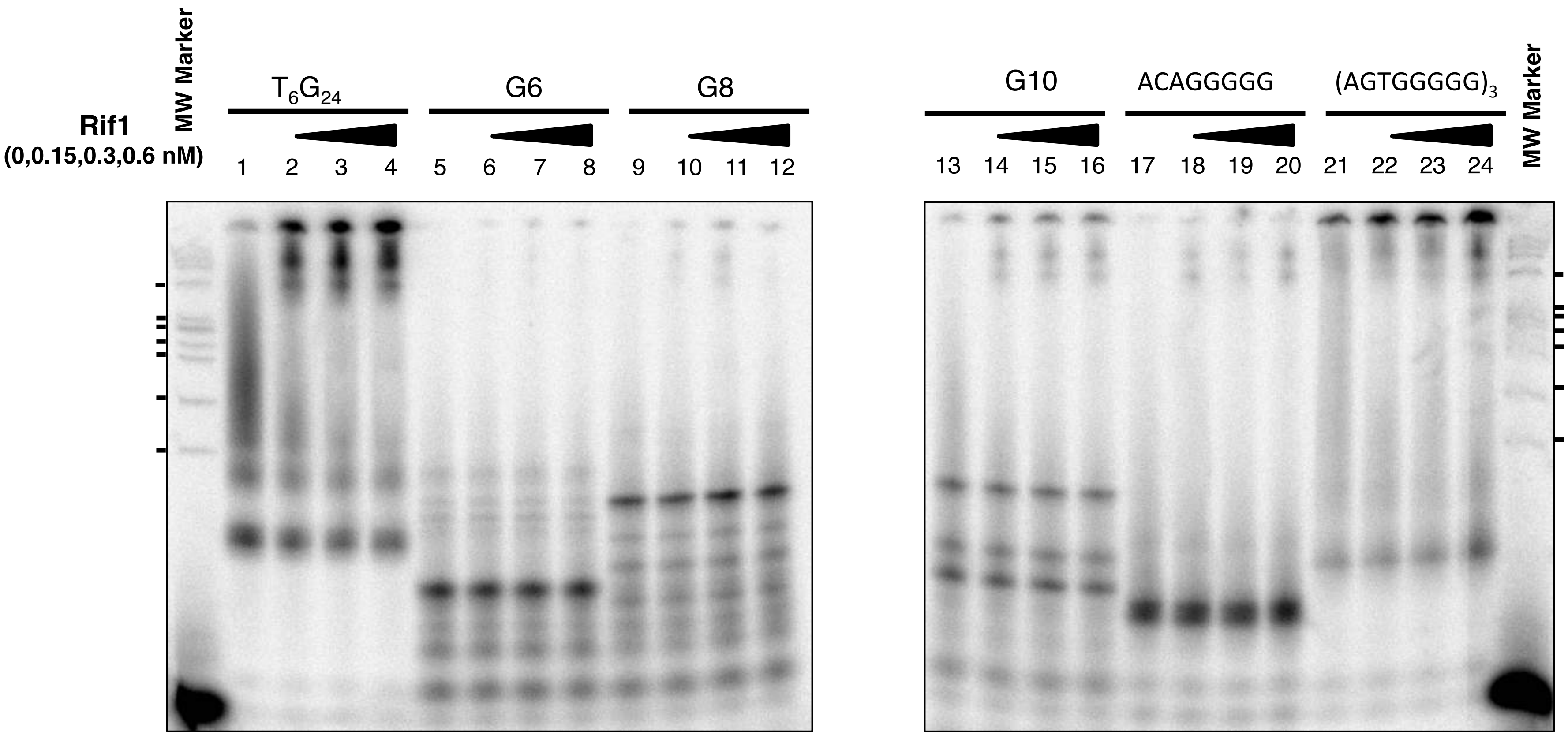

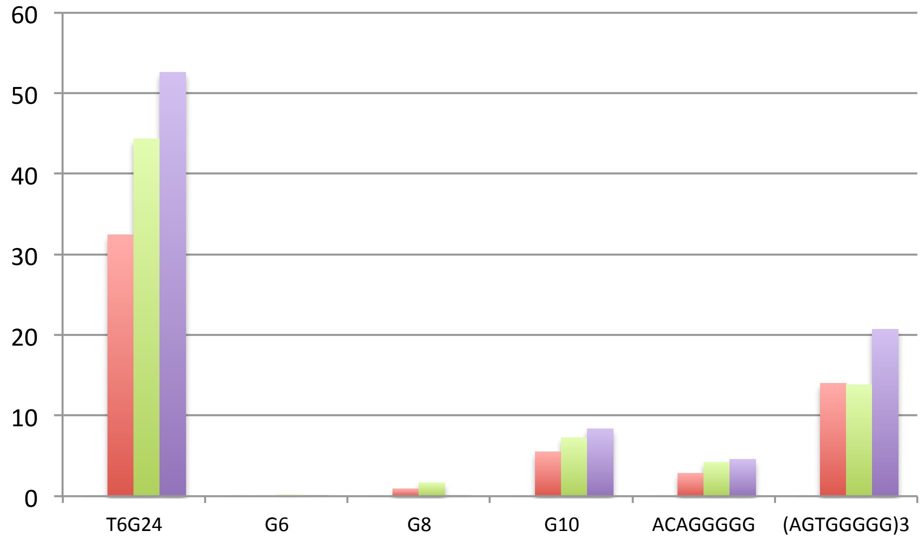

Supplementary Figure S7.
Binding of Rif1 to small G-tract oligonucleotides.
The single-stranded oligonucleotide DNAs (0.25 pmole) as shown, which had been heat-denatured and renatured in 50 mM KCl and 40% PEG200, were incubated with increasing amounts of the Rif1 full-length polypeptide, as shown in the figure. Samples were analyzed on 12% PAGE (1x TBE, 50 mM KCl and 40% PEG200). The ticks represent the sizes of 600, 310, 271/281, 234, 194, 118 and 72 bp, from the top.

Supplementary Figure S7

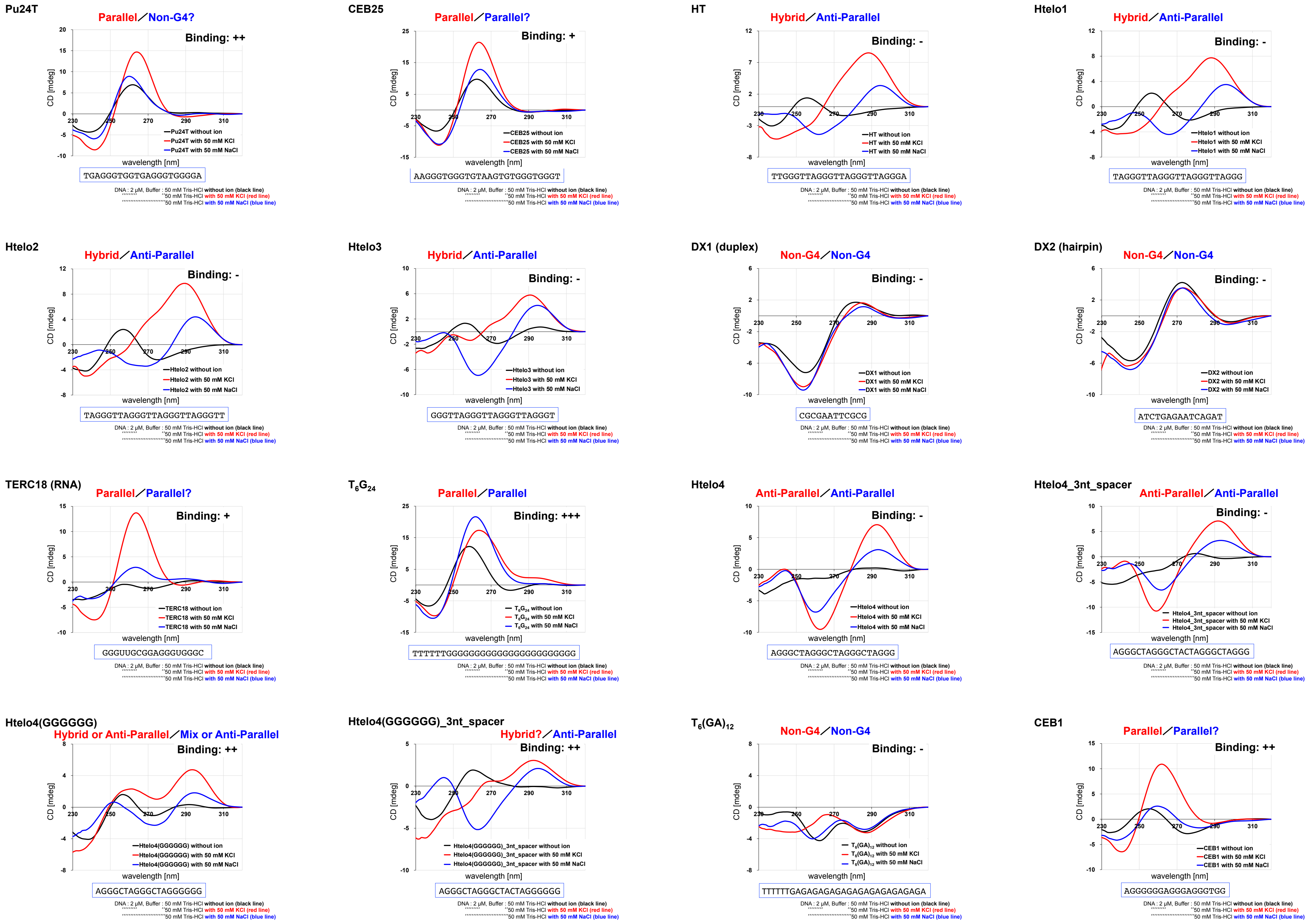

Supplementary Figure S7

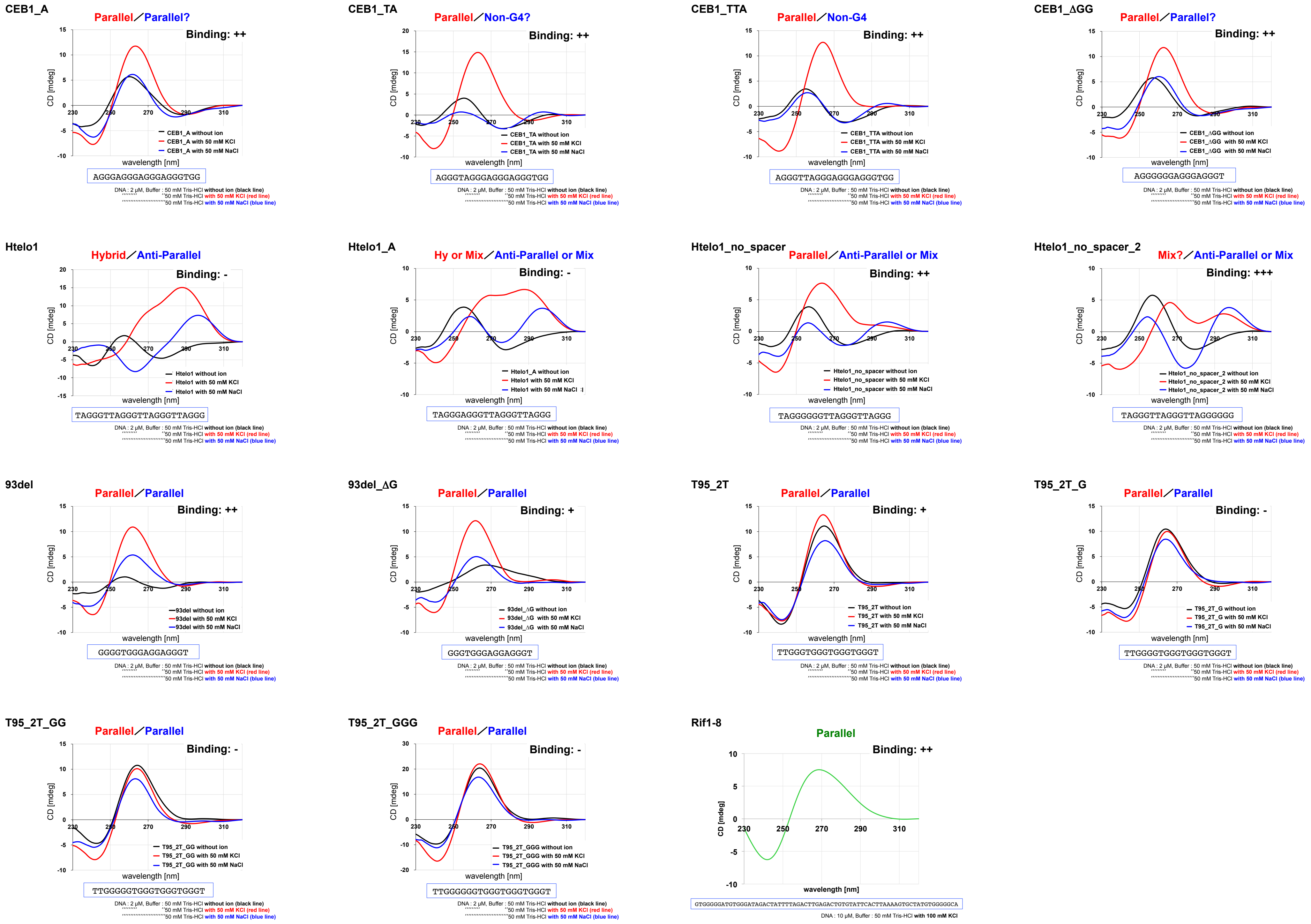

Supplementary Figure S8.

CD patterns of the oligonucleotides analyzed in this study.

CD measurement was conducted at 2μM DNA in 50 mM Tris-Cl (pH 7.5) without salt (black) or in 50 mM Tris-Cl (pH 7.5) containing 50 mM KCl (red) or 50 mM NaCl (blue). The sequence and relative binding affinity to Rif1 in the presence of KCl are also indicated for each oligonucleotide (-, +, ++, +++). The criteria of binding affinity is defined in the legend to Fig. 3. The topology of DNA estimated form the CD pattern is also presented in red (50 mM KCl) or in blue (50 mM NaCl). Summary of this measurement is shown in **Supplementary Table S1**.

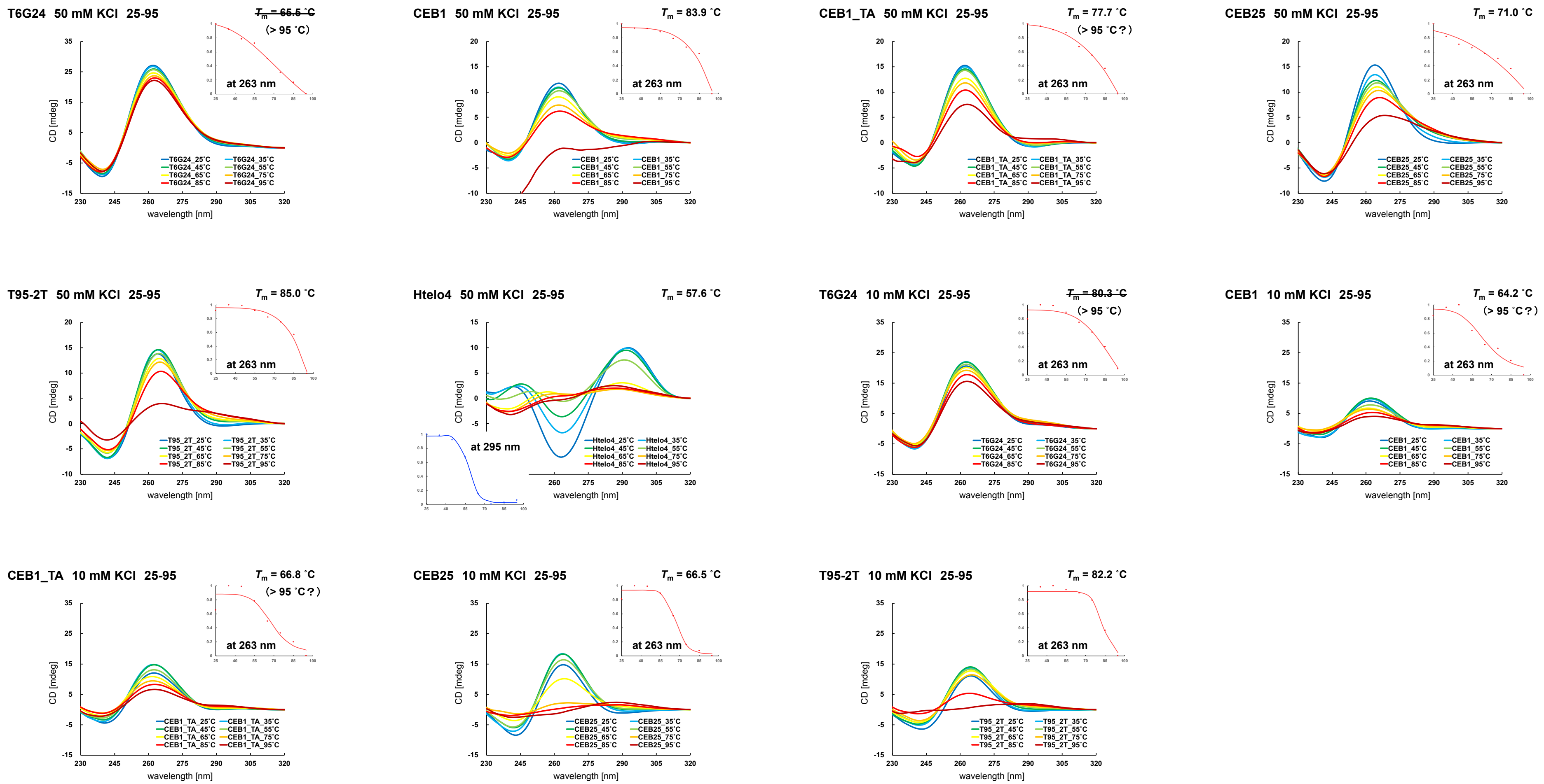

T6G24 : TTTTTTGGGGGGGGGGGGGGGGGGGGGGGG
CEB1 : AGGGGGGAGGGAGGGTGG
CEB1_TA : AGGGTAGGGAGGGAGGGTGG
CEB25 : AAGGGTGGGTGTAAGTGTGGGTGGGT
T95_2T : TTGGGTGGGTGGGTGGGT
Htelo4 : AGGGCTAGGGCTAGGGCTAGGG

Summary

		50 mM KCl	10 mM KCl	
	Topology	T_m (˚C)	T_m (˚C)	Binding
**T6G24**	Parallel	**> 95**	**> 95**	+++
**CEB1**	Parallel	83.9 (> 95)	64.2 (> 95)	++
**CEB1_TA**	Parallel	77.7 (> 95)	66.8 (> 95)	++
**CEB25**	Parallel	71.0	66.5	+
**T95_2T**	Parallel	85.0	82.2	+
**Htelo4**	Anti-parallel	57.6	57.6	-

Supplementary Figure S9.
Determination of melting temperatures of selected G4-forming sequences used in this study.
CD profiles were measured at various temperatures for a selected set of G4-forming sequences (with 50 mM and 10 mM KCl), as described in the legend to Supplementary Fig. S8. Tm for each sequence was estimated from the melting curve. CEB1 and CEB1_TA are capable of maintaining the parallel form even at 95°C, and thus their Tm are probably higher than 95°C.

Masai *et al*. Supplementary Figure S9

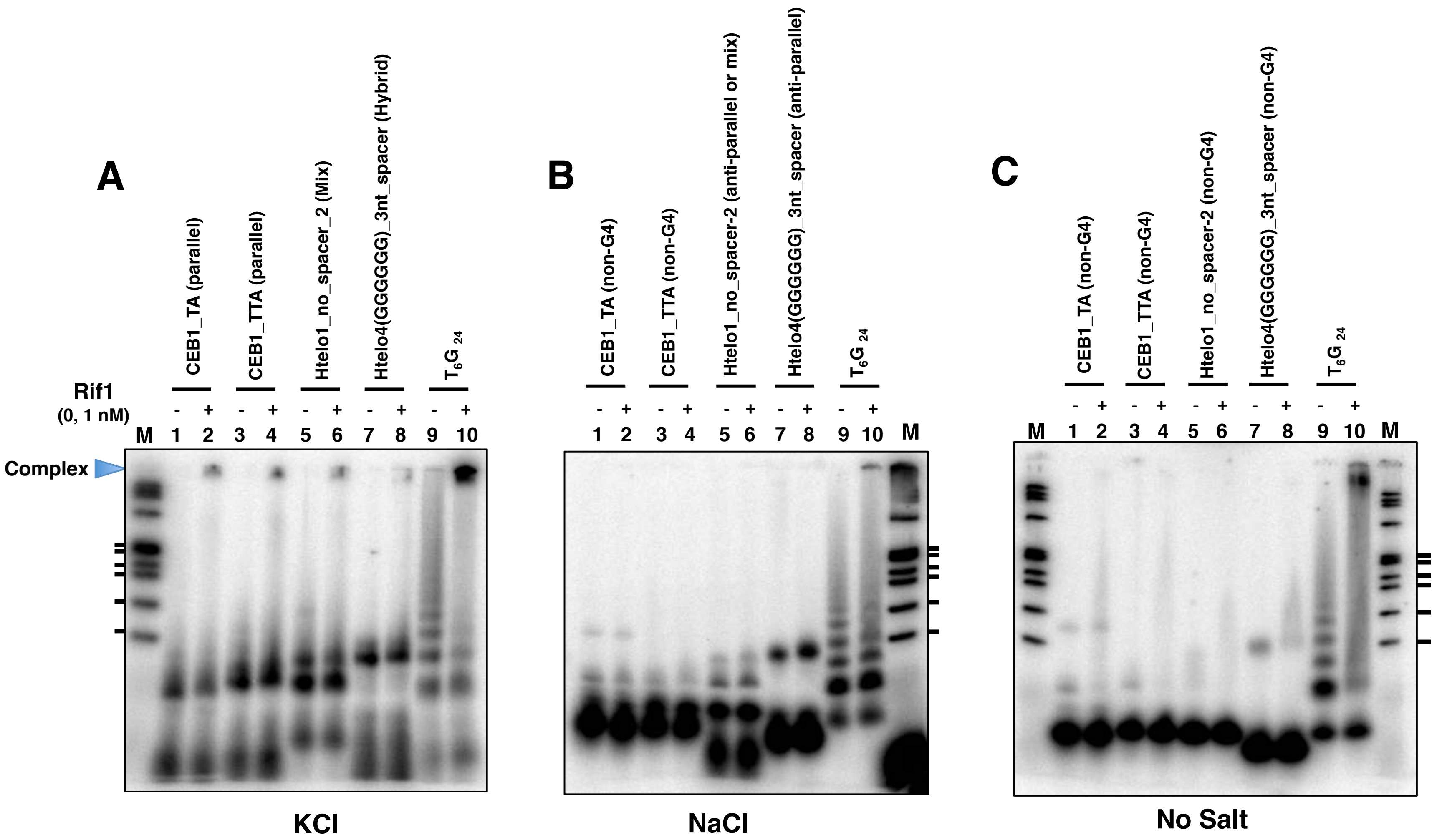

Supplementary Figure S10 (related to Fig. 5).
Effect of salt on Rif1 binding to selected G4 oligonucleotides whose topologies change in response to salt (another one of the triplicate experiments shown in Fig. 5).
The results of an independent set of assays identical to Fig. 5 are shown.

CEB1_TA:AGGGTAGGGAGGGAGGGTGG
CEB1_TTA:AGGGTTAGGGAGGGAGGGTGG
Htelo1_no_spacer_2:TAGGGTTAGGGTTAGGGGGG
Htelo4(GGGGGG)_3nt_spacer:AGGGCTAGGGCTACTAGGGGGG
T_6G_{24}:TTTTTTGGGGGGGGGGGGGGGGGGGGGGGG

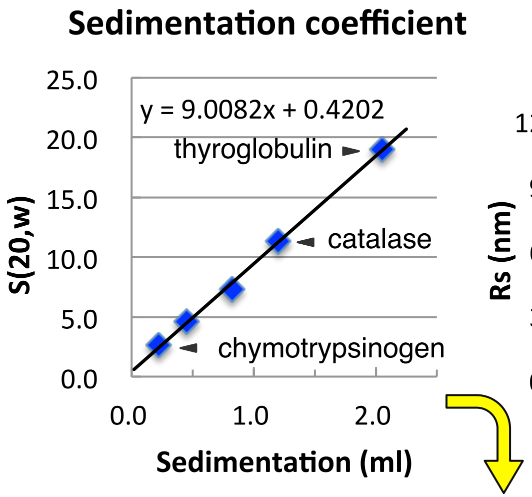

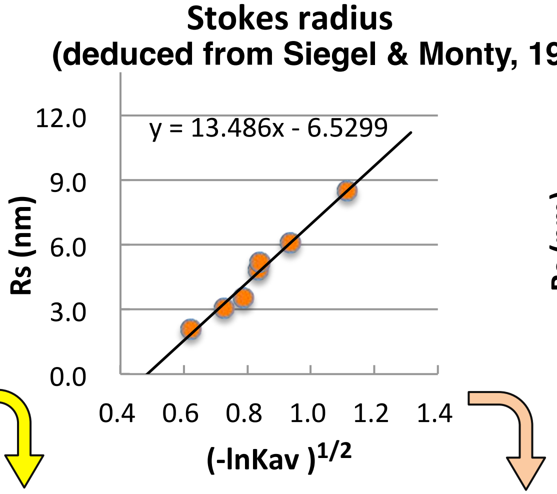

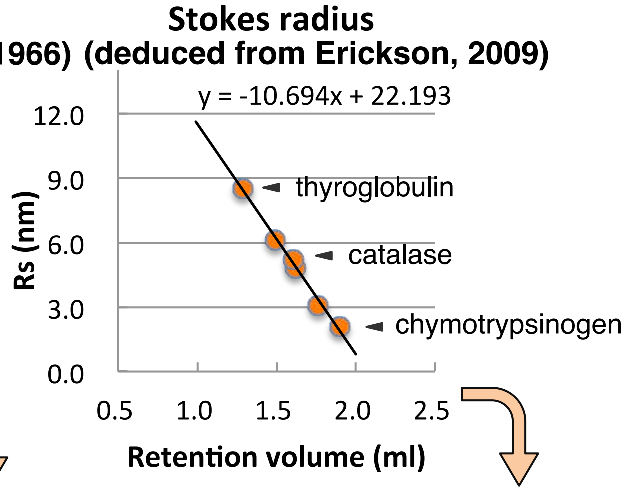

	Monomer Mw.	sediment (ml)	S value
Full–length Rif1(S)	161,332	1.250	13.129
Full–length Rif1(M)	161,332	1.650	17.404
Full–length Rif1(F)	161,332	2.050	21.679
70 kD polypeptide(S)	70,000	0.350	3.510
70 kD polypeptide(M1)	70,000	0.850	8.854
70 kD polypeptide(M2)	70,000	1.420	14.946
70 kD polypeptide(F)	70,000	2.050	21.679

retention (ml)	Kav	Rs (nm)	Native Mw.	Multimer	Smax/S
0.840	0.0063694	24.483	1,351,555	8.38	3.36
0.840	0.0063694	24.483	1,791,672	11.11	3.06
0.840	0.0063694	24.483	2,231,789	13.83	2.84
1.296	0.2968153	8.512	125,612	1.79	2.58
1.296	0.2968153	8.512	316,885	4.53	1.90
1.296	0.2968153	8.512	534,935	7.64	1.59
1.296	0.2968153	8.512	775,939	11.08	1.41

Rs (nm)	Native Mw.	Multimer	Smax/S
13.368	737,961	4.57	2.25
13.368	978,268	6.06	2.04
13.368	1,218,576	7.55	1.90
8.398	123,927	1.77	2.56
8.398	312,634	4.47	1.88
8.398	527,759	7.54	1.58
8.398	765,530	10.94	1.39

$$\textit{Native Mw.} = SN_0(6\pi\eta R_s)/(1-\nu_2\rho) = 4{,}205(SR_s)$$

Mw. = Native Molecular weight (Da)
S = Sedimentation coefficient (Svedverg unit = 10^{-13} s)
Rs = Stokes radius (nm)
N_0 = 6.023×10^{23} (Avogadro's number)
η = 0.01 g/cm·s (viscosity of the solvent, H_2O)
ν_2 = 0.73 cm^3/g (partial specific volume of most proteins)
ρ = 1.0 g/cm^3 (density of the solvent, H_2O)

$S_{max}/S = R/R_{min}$
1.2〜1.3: globular protein
1.5〜1.9: moderately elongated
2.0〜3.0: highly elongated
>3.0: very long thread-like molecule

Supplementary Figure S11. Calculation of native molecular weights of the full-length Rif1 and the ~70kDa degraded polypeptide. The values for both *S* (sedimentation coefficient; *left-most graph and table*) and *Rs* (Stokes radius) were determined. *Rs* was determined according either to Siegel and Monty (*middle graph and middle table*; ref. 1) or to Erickson (*right graph and right table*; ref 2). The native molecular weight (Native Mw.) and the oligomeric state (Multimer), calculated with the equation below the table (left side), are presented in the middle and right tables. *Smax/S* values were also calculated and are presented in the same tables. General interpretation of *Smax/S*, indicative of molecular shape, is given in a box below the tables.

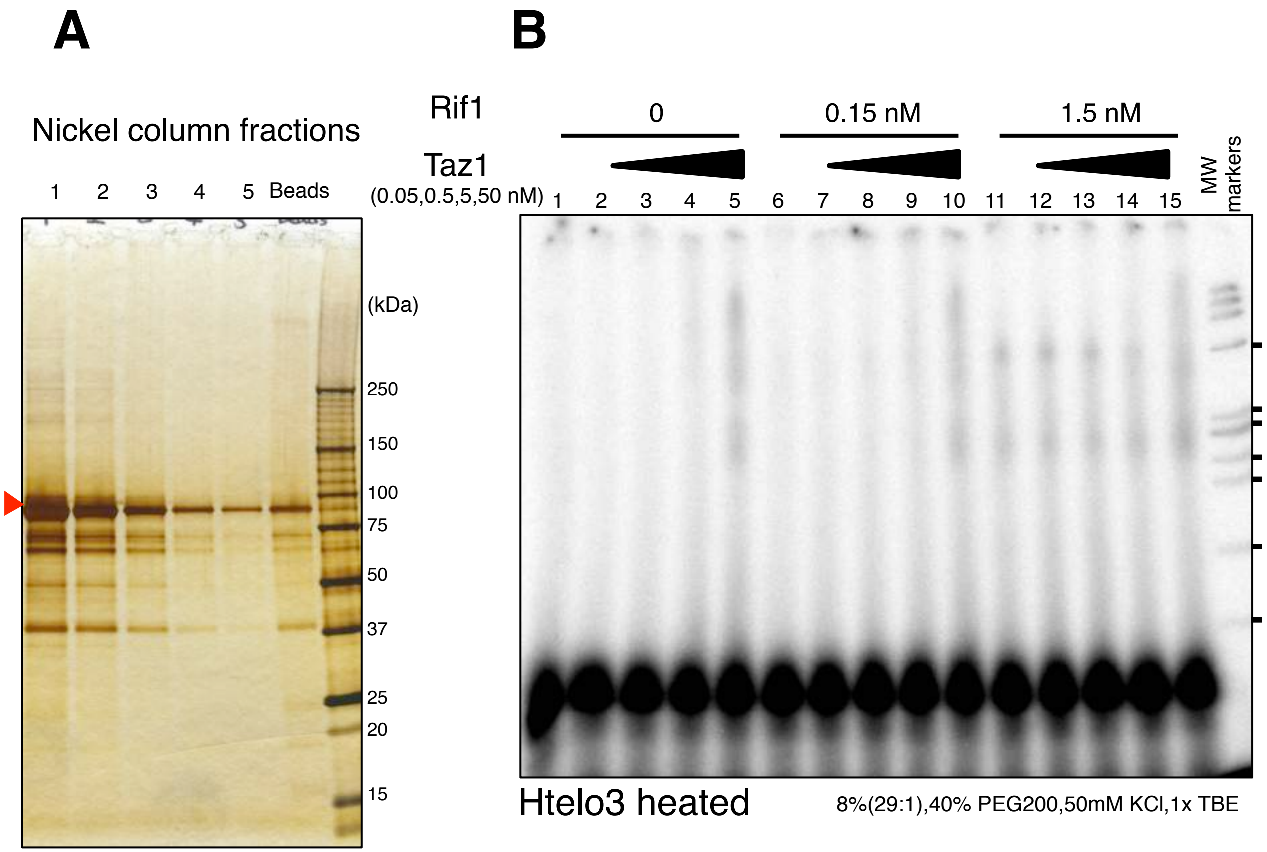

Htelo3:GGGTTAGGGTTAGGGTTAGGGT

Supplementary Figure S12

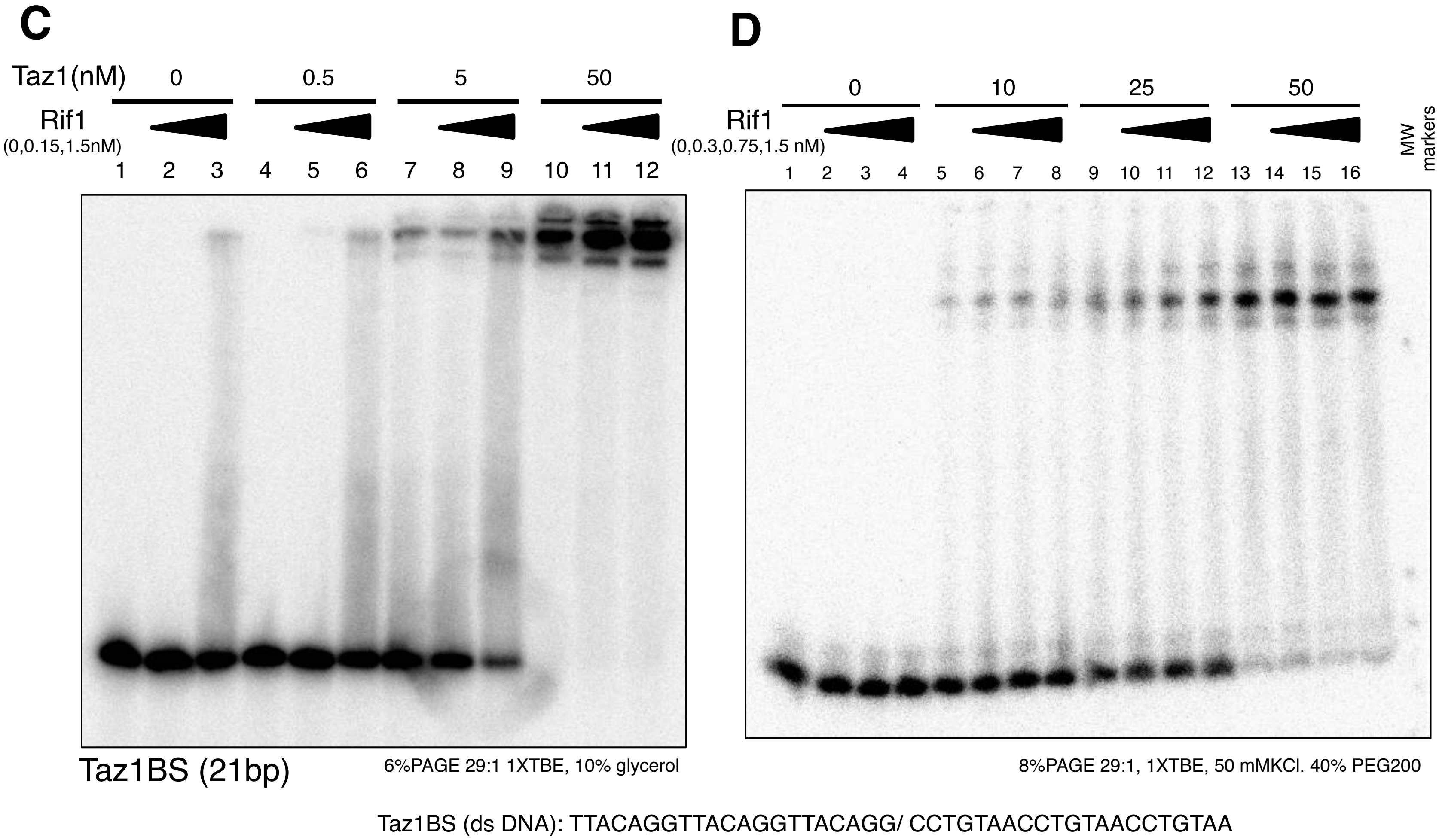

Supplementary Figure S12.
Effect of Taz1 on interaction of Rif1 with DNA

A. Purification of fission yeast Taz1 protein (663 aa). His_6-Rif1-$Flag_3$ protein expressed in human embryonic kidney 293T cells was purified by consecutive anti-Flag and nickel columns. Eluates fom nickel column were analyzed on SDS-PAGE (5-20% gradient gel) and stained by silver. **B**. ^{32}P-end labeled Htelo3 DNA (0.25 pmole) was mixed with the combination of Taz1 and Rif1 proteins, as indicated, and was analyzed on 8% PAGE (29:1; 1x TBE, 40% 50 mM KCl and 1 PEG200). **C**. and **D**. ^{32}P-end labeled Taz1BS dsDNA (0.25 pmole) was mixed with the combination of Taz1 and Rif1 proteins, as indicated, and was analyzed on 6% PAGE (29:1; 1xTBE and 10% glycerol) (C) or 8%PAGE (29:1; 1xTBE, 50 mM KCl, and 40% PEG200) (D). Ticks in the fX174/ HaeIII markers (B) represent 600, 310, 271/281, 234, 194, 118 and 72 bp.

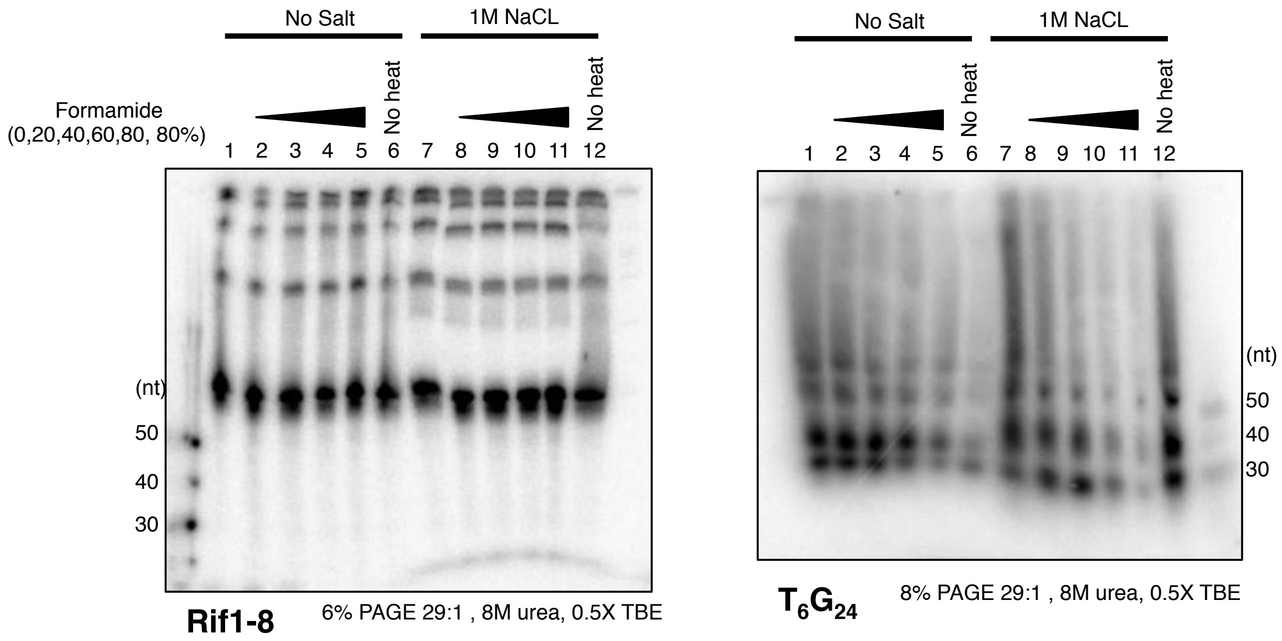

Rif1-8(70):GTGGGGGATGTGGGATAGACTATTTAGACTTGAGACTGTGTATTCACTTAAAGTGCTATGTGGGGGCA

T_6G_{24}(30):TTTTTTGGGGGGGGGGGGGGGGGGGGGGGG

Supplementary Figure S13.

Ladder formation in denaturing PAGE.

A.^{32}P-labeled Rif1-8 (0.25 pmole), denatured and reannealed in 100 mM KCl and 40% PEG200, were run on 6 % PAGE (29:1) with 8M urea in 0.5x TBE, and autoradiographed. **B.** ^{32}P-labeled T_6G_{24} (0.25 pmole), denatured and reannealed in 100 mM KCl and 40% PEG200, were run on 8 % PAGE (29:1) with 8M urea in 0.5x TBE, and autoradiographed. In both A and B, DNAs were pretreated with different concentration of formamide in the absence (lanes 1-6) or presence (lanes 7-12) of 1M NaCl. DNAs were heated at 96°C for 1 min before loading onto PAGE (lanes 1-5, 7-11). Ladders are still generated in a denaturing gel after heat denaturation in formamide, suggesting that the structures may not be simply stacked oligomers, but could include intertwined G4 structures made of multiple DNA strands.

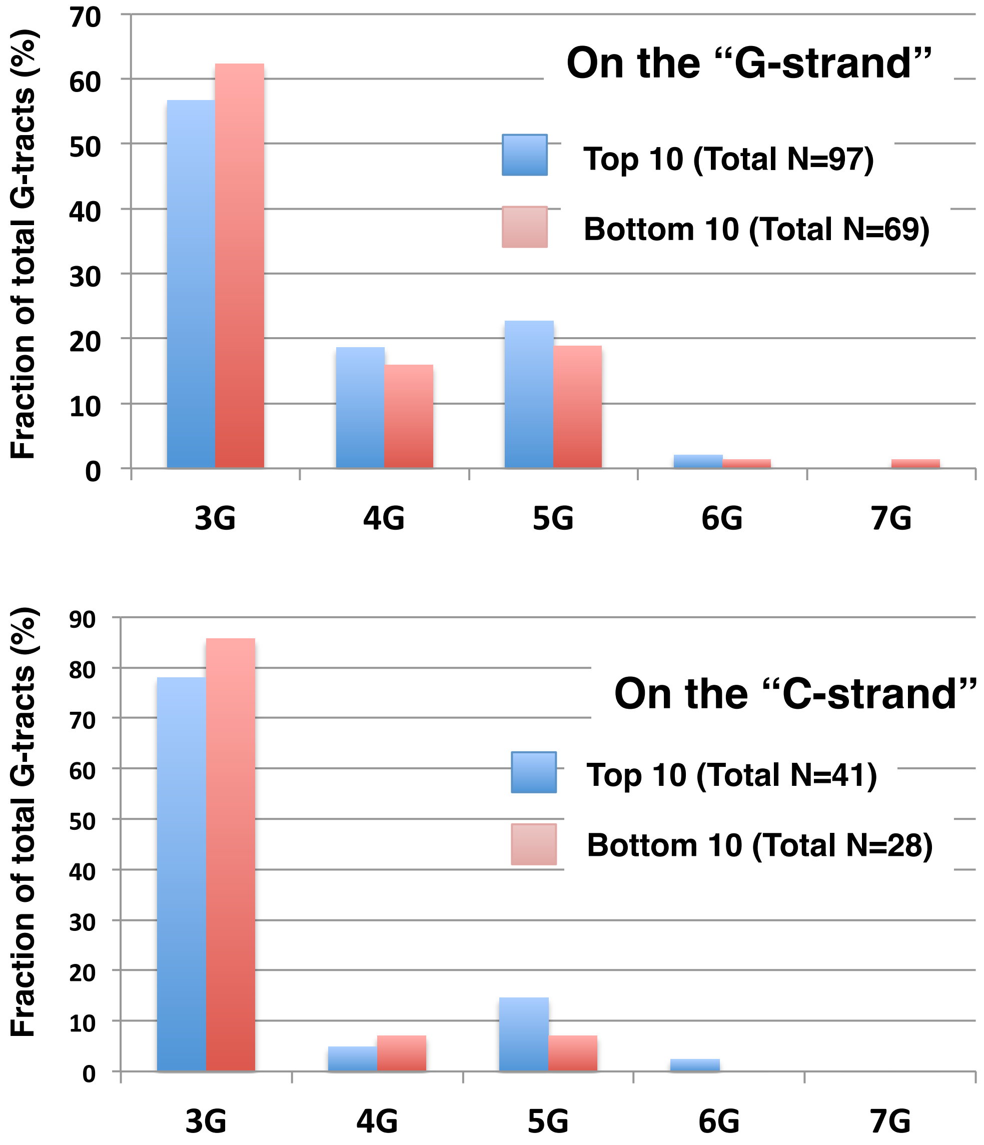

Supplementary Figure S14.
The length of G-tracts present in Rif1BS.
The sequences of the 1 kb segments encompassing the 35 Rif1BS were analyzed. All the G-tracts equal or longer than 3 were extracted, and the numbers of 3G~7G were scored for the "top 10" and "bottom 10" Rif1BS. The analyses were conducted for both strands. The G-strand represents the strand on which the G-tracts of Rif1CS are present, and the C-strand the other strand. Fractions of each G-tract are shown.

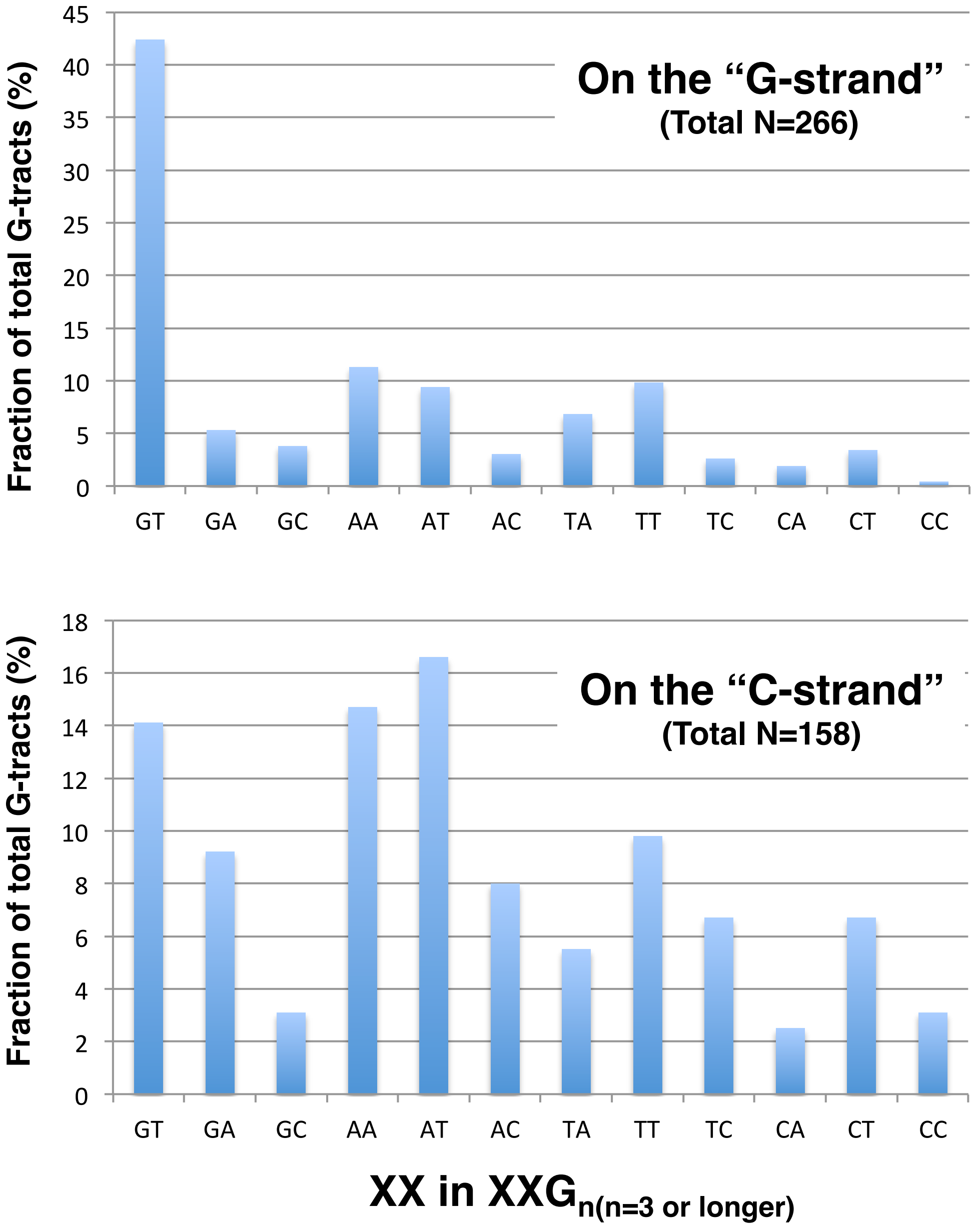

Supplementary Figure S15.
Frequency of various dinucleotide sequences preceding the G-tracts (n=3 or longer) in Rif1BS.
The sequences of the 1 kb segments encompassing the 35 Rif1BS were analyzed. All the G-tracts equal or longer than 3 were extracted, and the preceding dinucleotides were scored. The analysis was conducted on both strands. The G-strand represents the strand on which the G-tracts of Rif1CS are present, and the C-strand the other strand. Fractions of each dinucleotide are shown.

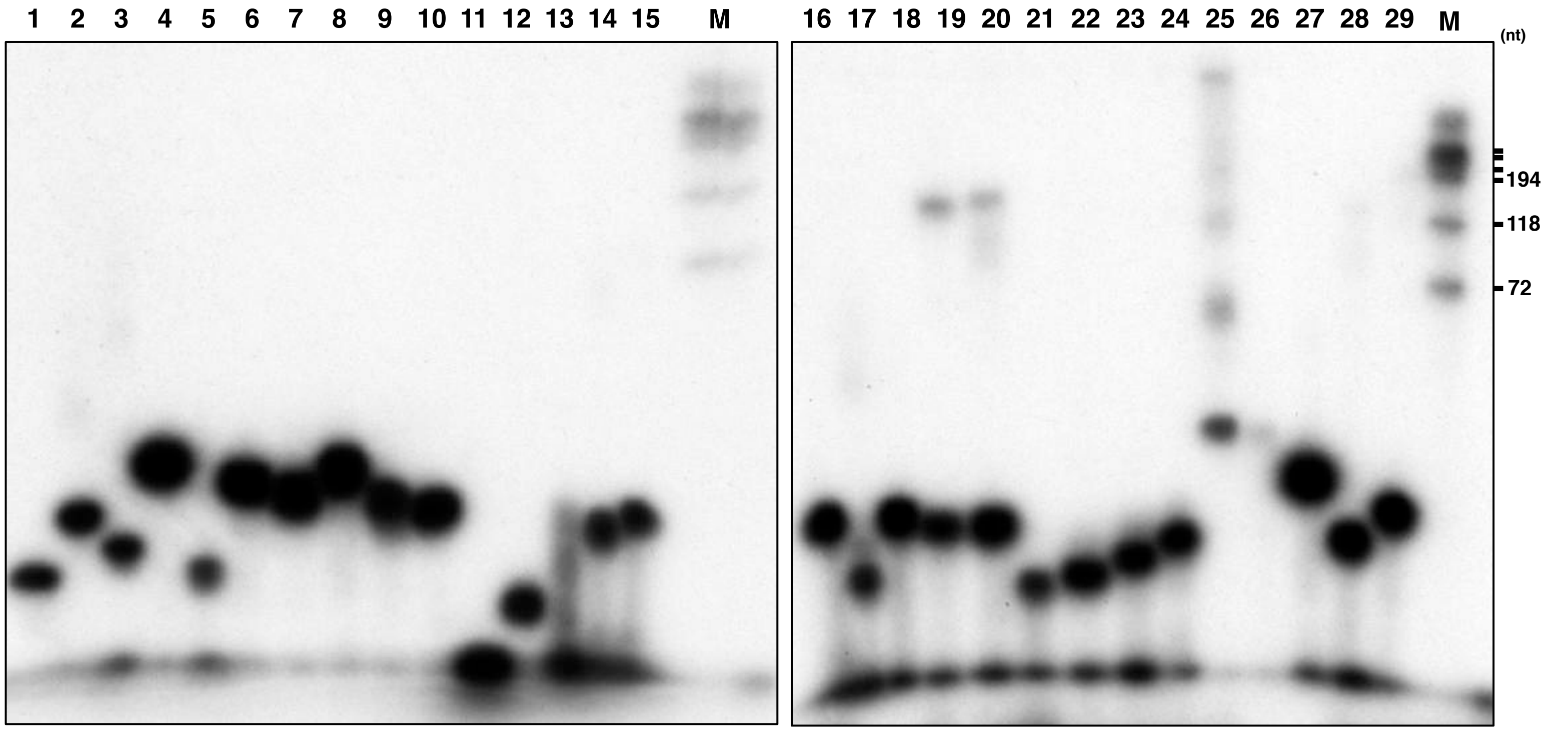

No.	Name	Sequence	Length (nt)
1	T95_2T	TTGGGTGGGTGGGTGGGT	18
2	Pu24T	TGAGGGTGGTGAGGGTGGGGA	21
3	CEB1	AGGGGGGAGGGAGGGTGG	18
4	CEB25	AAGGGTGGGTGTAAGTGTGGGTGGGT	26
5	93del	GGGGTGGGAGGAGGGT	16
6	HT	TTGGGTTAGGGTTAGGGTTAGGGA	25
7	Htelo1	TAGGGTTAGGGTTAGGGTTAGGG	23
8	Htelo2	TAGGGTTAGGGTTAGGGTTAGGGTT	25
9	Htelo3	GGGTTAGGGTTAGGGTTAGGGT	22
10	Htelo4	AGGGCTAGGGCTAGGGCTAGGG	22
11	DX1	CGCGAATTCGCG	12
12	DX2	ATCTGAGAATCAGAT	15
13	TERC18(RNA)	GGGUUGCGGAGGGUGGGC	18
14	CEB1_A	AGGGAGGGAGGGAGGGTGG	19
15	CEB1_TA	AGGGTAGGGAGGGAGGGTGG	20

No.	Name	Sequence	Length (nt)
16	CEB1_TTA	AGGGTTAGGGAGGGAGGGTGG	21
17	CEB1_ΔGG	AGGGGGGAGGGAGGGT	16
18	Htelo1_A	TAGGGAGGGTTAGGGTTAGGG	21
19	Htelo1_no_space	TAGGGGGGTTAGGGTTAGGG	20
20	Htelo1_no_space_2	TAGGGTTAGGGTTAGGGGGG	20
21	93del_ΔG	GGGTGGGAGGAGGGT	15
22	T95_2T_G	TTGGGGTGGGTGGGTGGGT	19
23	T95_2T_GG	TTGGGGGTGGGTGGGTGGGT	20
24	T95_2T_GGG	TTGGGGGGTGGGTGGGTGGGT	21
25	T_6G_{24}	TTTTTTGGGGGGGGGGGGGGGGGGGGGGGG	30
26	$T_6(GA)_{12}$	TTTTTTGAGAGAGAGAGAGAGAGAGAGAGA	30
27	Htelo4_3nt_pacer	AGGGCTAGGGCTACTAGGGCTAGGG	25
28	Htelo4(GGGGGG)	AGGGCTAGGGCTAGGGGGG	19
29	Htelo4(GGGGGG)_3nt_spacer	AGGGCTAGGGCTACTAGGGGGG	22

Supplementary Figure S16. Analyses of labeled oligonucleotides on denaturing PAGE containing 8M urea. Oligonucleotides used in this studies were end-labeled by T4 polynucleotide kinase, purified by gel filtration, (spun column) denatured in 95% formamide containing 5 mM EDTA and 20 mM LiCl, and were run on 15% PAGE containing 8M urea in 0.5x TBE. T_6G_{24} appears as a ladder, since it forms secondary structures even in the presence of 8M urea. The bands at the bottom of the gels are free labeled nucleotides that remained after gel filtration purification of labeled oligonucleotides. In lane11, the oligonucleotide (12mer) was too small to be separated on this gel.

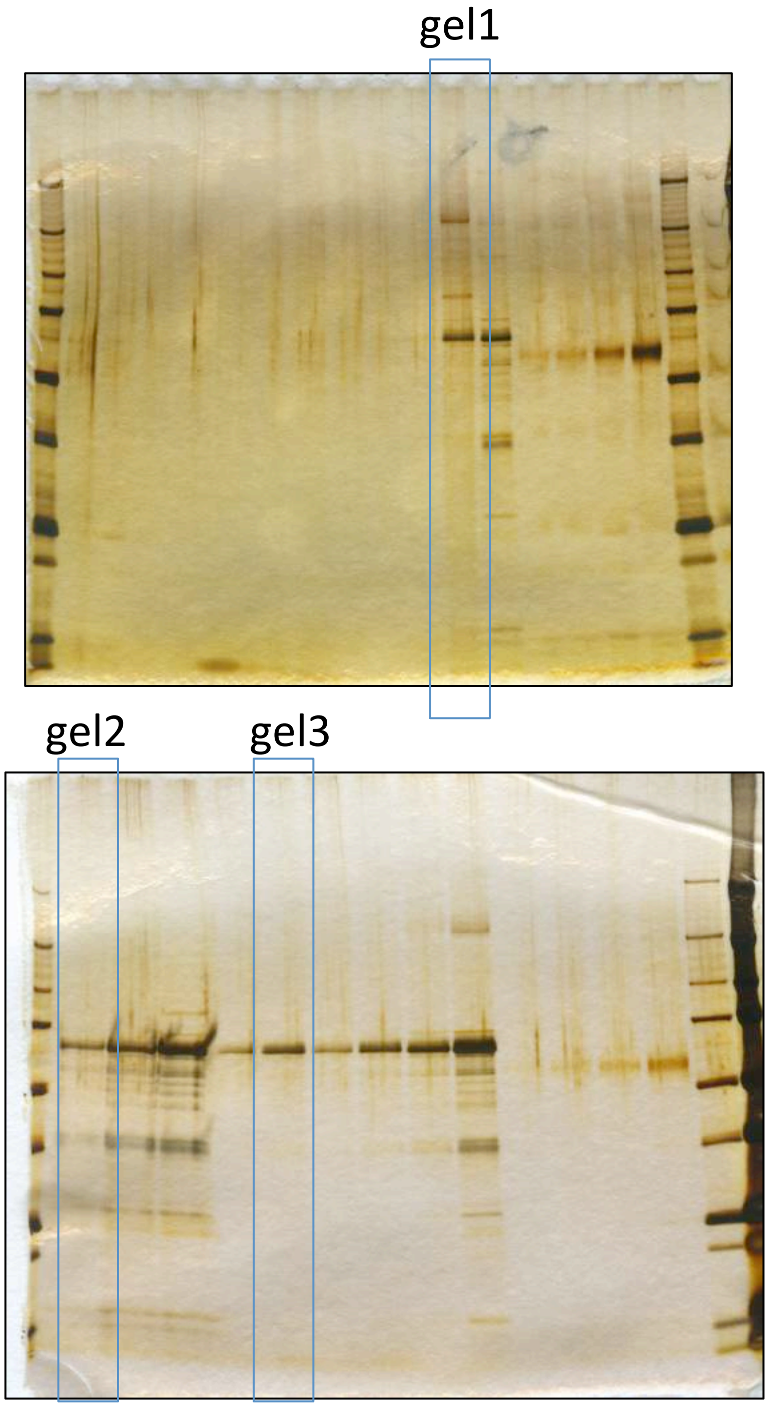

Supplementary Figure S17. Original full-length gels for data in Supplementary Figure S1.
Figure S1A lane 1: gel1
Figure S1A lane 2: gel2
Figure S1A lane 3: gel3

Name	Sequence	Structure	Topology	References
CEB1	AGGGGGGAGGGA GGGTGG	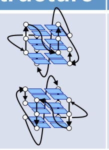	Parallel-type (dimeric form)	Adrian, M., Ang, D. J., Lech, C. J., Heddi, B., Nicolas, A. & Phan, A. T. Structure and conformational dynamics of a stacked dimeric G-quadruplex formed by the human CEB1 minisatellite. J. Am. Chem. Soc. 136, 6297-6305 (2014).
CEB25	AAGGGTGGGTGTA AGTGTGGGTGGGT	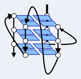	Parallel-type	Amrane, S., Adrian, M., Heddi, B., Serero, A., Nicolas, A., Mergny, J. L. & Phan, A. T. Formation of pearl-necklace monomorphic G-quadruplexes in the human CEB25 minisatellite. J. Am. Chem. Soc. 134, 5807-5816 (2012).
T95_2T	TTGGGTGGGTGGG TGGGT	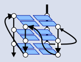	Parallel-type	Do, N. Q. & Phan, A. T. Monomer-dimer equilibrium for the 5'-5' stacking of propeller-type parallel-stranded G-quadruplexes: NMR structural study. Chem. Eur J. 18, 14752-14759 (2012).
Pu24T (*c-myc*)	TGAGGGTGGTGAG GGTGGGGA(AGG)	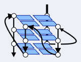	Parallel-type	Chung, W. J., Heddi, B., Hamon, F., Teulade-Fichou, M. P. & Phan, A. T. Solution structure of a G-quadruplex bound to the bisquinolinium compound Phen-DC(3). Angew. Chem. Int. Ed. 53, 999-1002 (2014).<br>Phan, A. T., Kuryavyi, V., Gaw, H. Y. & Patel, D. J. Small-molecule interaction with a five-guanine-tract G-quadruplex structure from the human MYC promoter. Nat. Chem. Biol. 1, 167-173 (2005).<br>Heddi, B., Cheong, V. V., Martadinata, H. & Phan, A. T. Insights into G-quadruplex specific recognition by the DEAH-box helicase RHAU: Solution structure of a peptide-quadruplex complex. Proc. Natl. Acad. Sci. U. S. A. 112, 9608-9613 (2015).
93del (aptamer)	GGGGTGGGAGGAG GGT	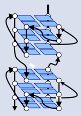	Parallel-type (dimeric form)	Phan, A. T., Kuryavyi, V., Ma, J. B., Faure, A., Andreola, M. L. & Patel, D. J. An interlocked dimeric parallel-stranded DNA quadruplex: a potent inhibitor of HIV-1 integrase. Proc. Natl. Acad. Sci. U. S. A. 102, 634-639 (2005).
HT (Human telomere)	TTGGGTTAGGGTT AGGGTTAGGGA	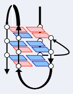	Hybrid-type (form 1)	Chung, W. J., Heddi, B., Tera, M., Iida, K., Nagasawa, K. & Phan, A. T. Solution structure of an intramolecular (3 + 1) human telomeric G-quadruplex bound to a telomestatin derivative. J. Am. Chem. Soc. 135, 13495-13501 (2013).<br>Luu, K. N., Phan, A. T., Kuryavyi, V., Lacroix, L. & Patel, D. J. Structure of the human telomere in K+ solution: an intramolecular (3 + 1) G-quadruplex scaffold. J. Am. Chem. Soc. 128, 9963-9970 (2006).
Htelo1 (Human telomere)	TAGGGTTAGGGTT AGGGTTAGGG	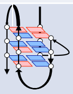	Hybrid-type (form 1)	Phan, A. T., Kuryavyi, V., Luu, K. N. & Patel, D. J. Structure of two intramolecular G-quadruplexes formed by natural human telomere sequences in K+ solution. Nucleic Acids Res. 35, 6517-6525 (2007).
Htelo2 (Human telomere)	TAGGGTTAGGGTT AGGGTTAGGGTT	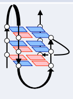	Hybrid-type (form 2)	Phan, A. T., Kuryavyi, V., Luu, K. N. & Patel, D. J. Structure of two intramolecular G-quadruplexes formed by natural human telomere sequences in K+ solution. Nucleic Acids Res. 35, 6517-6525 (2007).
Htelo3 (Human telomere)	GGGTTAGGGTTAG GGTTAGGGT	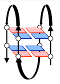	Anti-parallel-type	Lim, K. W., Amrane, S., Bouaziz, S., Xu, W., Mu, Y., Patel, D. J., Luu, K. N. & Phan, A. T. Structure of the human telomere in K+ solution: a stable basket-type G-quadruplex with only two G-tetrad layers. J. Am. Chem. Soc. 131, 4301-4309 (2009).
Htelo4 (Human telomere)	AGGGCTAGGGCTA GGGCTAGGG	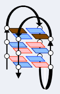	Chair-type	Lim, K. W., Alberti, P., Guedin, A., Lacroix, L., Riou, J. F., Royle, N. J., Mergny, J. L. & Phan, A. T. Sequence variant (CTAGGG)n in the human telomere favors a G-quadruplex structure containing a G.C.G.C tetrad. Nucleic Acids Res. 37, 6239-6248 (2009).
TERC18 (Human Telomerase RNA (hTERC))	GGGUUGCGGA GGGUGGGC	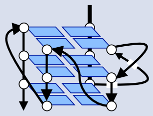	Parallel-type (proposed) + dimer (in equilibrium)	Martadinata, H. & Phan, A. T. Formation of a Stacked Dimeric G-Quadruplex Containing Bulges by the 5′ -Terminal Region of Human Telomerase RNA (hTERC). Biochemistry 53, 1595-1600 (2014).
G_{15}	GGGGGGGGGG GGGGG	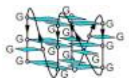	a propeller-type parallel-stranded G-quadruplex	Sengar, A., Heddi, B. and Phan, A.T. Formation of G-quadruplexes in poly-G sequences: structure of a propeller-type parallel-stranded G-quadruplex formed by a G_{15} stretch. (2014) *Biochemistry* **53**, 7718-7723.
